# An activity-based probe for antimicrobial target DXP synthase, a thiamin diphosphate-dependent enzyme

**DOI:** 10.3389/fchbi.2024.1389620

**Published:** 2024-05-07

**Authors:** Lauren B. Coco, Caren L. Freel Meyers

**Affiliations:** Department of Pharmacology and Molecular Sciences, Johns Hopkins University School of Medicine, Baltimore, MD, United States

**Keywords:** 1-deoxy-D-xylulose 5-phosphate synthase, activity-based probe, alkyl acetylphosphonate, thiamin-dependent enzyme, antimicrobial target, vitamin biosynthesis, isoprenoid biosynthesis, bacterial central metabolism

## Abstract

This work reports an alkyl acetylphosphonate (alkylAP) activity-based probe (ABP) for 1-deoxy-d-xylulose 5-phosphate synthase DXPS, a promising antimicrobial target. This essential thiamin diphosphate (ThDP)-dependent enzyme operates at a branchpoint in bacterial central metabolism and is believed to play key roles in pathogen adaptation during infection. How different bacterial pathogens harness DXPS activity to adapt and survive within host environments remains incompletely understood, and tools for probing DXPS function in different contexts of infection are lacking. Here, we have developed alkylAP-based ABP **1**, designed to react with the ThDP cofactor on active DXPS to form a stable C2α-phosphonolactylThDP adduct which subsequently crosslinks to the DXPS active site upon photoactivation. ABP **1** displays low micromolar potency against DXPS and dose-dependent labeling of DXPS that is blocked by alkylAP-based inhibitors. The probe displays selectivity for DXPS over ThDP-dependent enzymes and is capable of detecting active DXPS in a complex proteome. These studies represent an important advance toward development of tools to probe DXPS function in different contexts of bacterial infection, and for drug discovery efforts on this target.

## 1 Introduction

Identification of novel antimicrobial targets is essential to address the continued global threat of antimicrobial resistance (World Health Organization, 2022). Bacterial central metabolism remains relatively underexplored, yet promising, for development of new antimicrobial strategies (Murima, McKinney, and Pethe, 2014; Tong and Brown, 2023). Targeting pathogen adaptation is a particularly intriguing facet of developing this target space. Bacteria undergo metabolic remodeling in response to fluctuations in nutrient availability within the host (Passalacqua, Charbonneau, and O’Riordan, 2016; Fuchs et al., 2012; L. Rohmer, Hocquet, and Miller, 2011; Alteri and Mobley, 2012). These so-called metabolic adaptations are pathogen-specific responses that are essential for survival and pathogenicity in particular host environments (Alteri and Mobley, 2015; Turner et al., 2015). Thus, targeting essential metabolic adaptations offers the potential for narrow-spectrum antimicrobial strategies that avoid toxicity to healthy microbiota.

**FIGURE 1:**
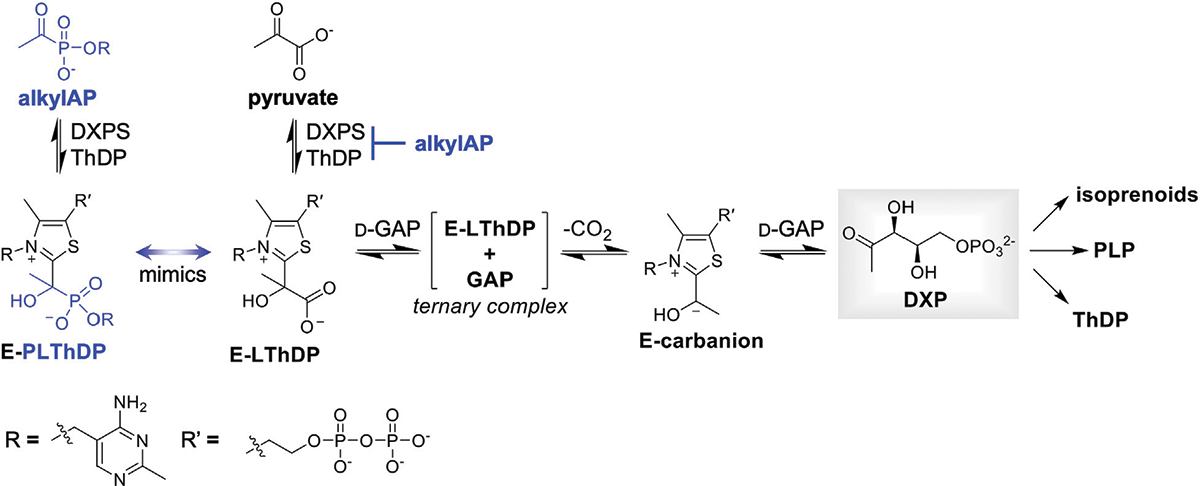
DXP is positioned at a branchpoint in bacterial metabolism and is required for synthesis of vitamins thiamin diphosphate (ThDP) and pyridoxal phosphate (PLP), and isoprenoids. DXPS catalyzes formation of DXP from pyruvate and d-GAP via a C2α-lactylThDP (LThDP) intermediate, and is inhibited by alkylAPs via formation of a stable phosphonolactylThDP (PLThDP) adduct.

1-Deoxy d-xylulose 5-phosphate synthase (DXPS) is an essential central metabolic enzyme that we hypothesize is critical for bacterial metabolic adaptation (Bartee D. and Freel Meyers CL. 2018; Sanders et al., 2017; E. C. Chen and Freel Meyers, 2023). This enzyme catalyzes the thiamin diphosphate (ThDP)-dependent formation of DXP from pyruvate and d-glyceraldehyde 3-phosphate (d-GAP). Absent in humans but widespread in high priority Gram-negative bacterial pathogens (Heuston et al., 2012; World Health Organization, 2022; Allamand et al., 2023), DXP is a branchpoint metabolite that serves as a precursor to vitamins B1 (ThDP) and B6 (pyridoxal phosphate, PLP), as well as isoprenoids biosynthesized via the methylerythritol phosphate (MEP) pathway (Figure 1) (Rodríguez-Concepción and Boronat, 2002; Hill, Sayer, and Spenser, 1989; M. Rohmer et al., 1993; David et al., 1981). Based on its role in these essential metabolic pathways, DXPS should be critical for pathogen adaptations that require vitamins or isoprenoids.

We have recently demonstrated such a role for DXPS in the adaptation of uropathogenic *Escherichia coli* (UPEC) to d-Ser, a bacteriostatic host metabolite present at high concentrations within the urinary tract (E. C. Chen and Freel Meyers, 2023). UPEC detoxify d-Ser through PLP-dependent conversion to pyruvate. Inhibiting DXPS sensitizes UPEC to d-Ser, and makes this pathogen vulnerable to inhibition of CoA biosynthesis in the context of urinary tract infection where the TCA cycle and gluconeogenesis from amino acids are critical for survival (Alteri, Smith, and Mobley, 2009; Alteri and Mobley, 2012, 2015; Alteri et al., 2019; Himpsl et al., 2020; Chan and Lewis, 2022). This is consistent with observations that bacterial sensitivity to DXPS inhibition depends upon the growth environment (Sanders et al., 2017, 2018), which suggests the degree to which bacteria rely on DXPS activity is context-dependent. Its interesting gated mechanism (Patel et al., 2012; Nemeria et al., 2009; Zhou et al., 2017; P. Y.-T. Chen et al., 2019; Toci et al., 2024; DeColli et al., 2019) and alternative activities (Brammer and Meyers, 2009; Morris et al., 2013; DeColli et al., 2018; Johnston and Freel Meyers, 2021; Johnston et al., 2022) also hint that DXPS may have other uncharacterized functions. If essential for a pathogen adaptation and survival during infection, such functions could potentially be targeted in an infection-specific manner.

Understanding the pathogen-specific roles of DXPS and/or contexts in which pathogens are highly sensitive to the loss of DXPS activity in one or more pathways at this metabolic branchpoint will help guide development of narrow-spectrum antibacterial strategies targeting DXPS. Paramount to this goal is access to tools that enable investigation of DXPS activity in different biological contexts. An activity-based probe (ABP) of DXPS would be particularly useful in this regard, as well as for discovery and development of antibacterial agents targeting DXPS. To our knowledge, there are currently no ABPs for DXPS or other ThDP-dependent enzymes. This study takes a first step toward development of ABPs for DXPS, drawing on our cumulative knowledge of DXPS mechanism and previous efforts to develop selective inhibitors.

In the first step of the reaction catalyzed by DXPS, pyruvate reacts with the ThDP cofactor to form a C2α-lactylthiamin diphosphate (LThDP) intermediate (Figure 1). Alkyl acetylphosphonate (alkylAP) inhibitors were designed as stable pyruvate mimics to study ThDP-dependent pyruvate decarboxylase enzymes (O’Brien et al., 1980; Kluger and Pike, 1977; R. Kluger and Tsui, 1986), and they react with ThDP in a similar manner to form a stable C2α-phosphonolactylthiamin diphosphate (PLThDP) adduct. We have advanced alkylAP inhibitor development to target the large DXPS active site and its gated mechanism requiring ternary complex formation (Smith, Vierling, and Meyers, 2012; Morris et al., 2013; Sanders et al., 2017; Bartee and Meyers, 2018a; Coco et al., 2024). Here, we describe the first activity-based probe for DXPS, ABP **1**, based on first-generation alkylAP inhibitors. Our results demonstrate dose-dependent labeling of DXPS by **1** via a mechanism involving PLThDP formation on active DXPS, and show that labeling is blocked in a concentration-dependent manner by DXPS inhibitors of varying potency. ABP **1** also displays selectivity for DXPS over ThDP-dependent pyruvate dehydrogenase E1 subunit (PDH) and pyruvate decarboxylase (PDC) *in vitro*, and detects active DXPS in a complex proteome. These studies represent an important advance toward development of tools to probe DXPS function in different contexts of bacterial infection, and for drug discovery efforts on this target.

## 2 Results

### 2.1 Design and synthesis of an activity-based probe for DXPS

**FIGURE 2:**
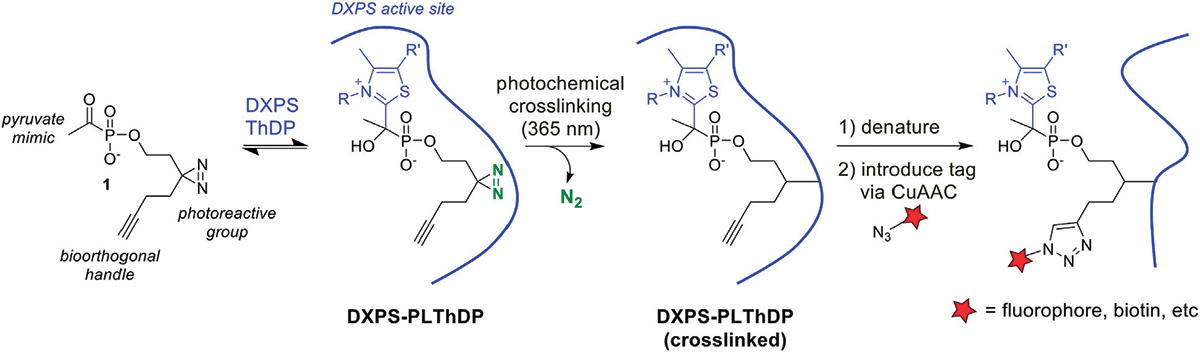
Activity-based probe design and workflow. PLThDP forms via a reversible reaction of **1** with ThDP in the DXPS active site. Upon irradiation, diazirine reacts to release N_2_ leaving behind a reactive carbene which irreversibly crosslinks the DXPS active site. Following crosslinking, DXPS is denatured and subjected to the CuAAC reaction to introduce a tag, enabling detection of labeled DXPS.

As a first step to develop ABPs for DXPS, we designed a probe based on the first-generation alkylAP scaffold (Figure 2). The probe design incorporates the acetylphosphonate reactive group that mimics the donor substrate pyruvate, and reacts with the ThDP cofactor on active DXPS to form the covalent PLThDP adduct. As PLThDP formation is reversible (Sanders et al., 2017), a crosslinking group is required to ensure that active DXPS can be labeled irreversibly. Sterically demanding substituents can be incorporated into the phosphonyl ester group without significant loss in inhibitor potency (Morris et al., 2013; Sanders et al., 2017; Bartee and Meyers, 2018a; Coco et al., 2024), due to the large active site volume of DXPS, whereas modifications to the reactive acetyl group are not tolerated (Smith, Vierling, and Meyers, 2012). Based on this, we designed alkylAP-based probe **1** bearing the commonly-used 2-(3-(but-3-yn-1-yl)-3H-diazirin-3-yl)ethyl moiety (Li et al., 2013), capable of crosslinking to the DXPS active site and presenting a biorthogonal handle for introduction of a fluorophore or biotin.

**FIGURE 3:**
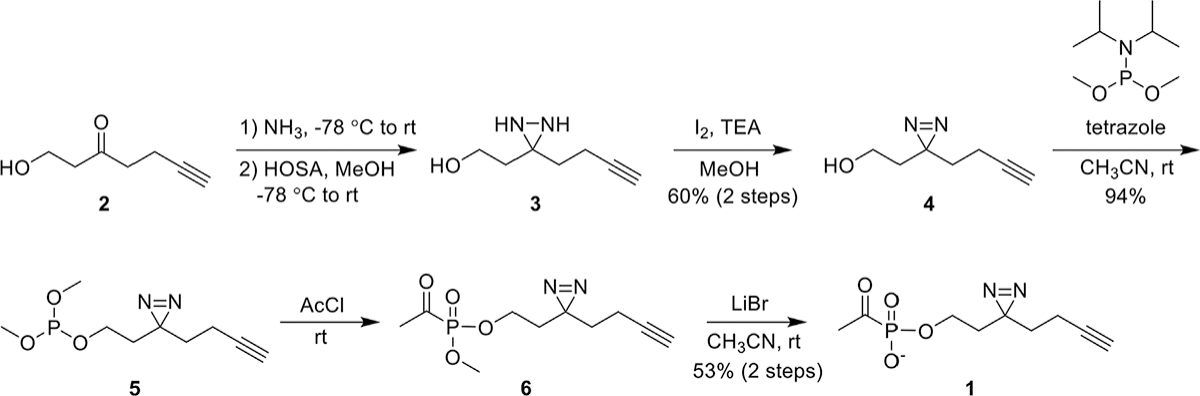
Synthesis of **1** from commercially available 1-hydroxy-6-heptyn-3-one **2** in 5 steps. *Abbreviations*: hydroxylamine-*O*-sulfonic acid (HOSA); triethylamine (TEA); acetyl chloride (AcCl).

ABP **1** was synthesized from the commercially available 1-hydroxy-6-heptyn-3-one **2** (Figure 3). Ketone **2** was converted to diaziridine **3** by sequential treatment with anhydrous ammonia and hydroxylamine-*O*-sulfonic acid (HOSA). Oxidation of crude **3** using iodine afforded diazirine **4** in 60% yield over two steps. Phosphorylation of **4** via phosphoramidite coupling with dimethyl-*N*,*N*-diisopropylphosphoramidite in the presence of tetrazole gave phosphite 5 in 94% yield. Phosphite **5** was converted to acetylphosphonate diester **6** by reaction with acetyl chloride, and **6** was subsequently dealkylated with lithium bromide to give ABP **1** in 53% yield over two steps. As expected, ABP **1** exhibited an absorbance profile consistent with a diazirine ring (λ_max_ 340 nm) (Supplementary Figure S1).

### 2.2 ABP 1 inhibits *E. coli* DXPS via formation of a phosphonolactylThDP adduct

**FIGURE 4:**
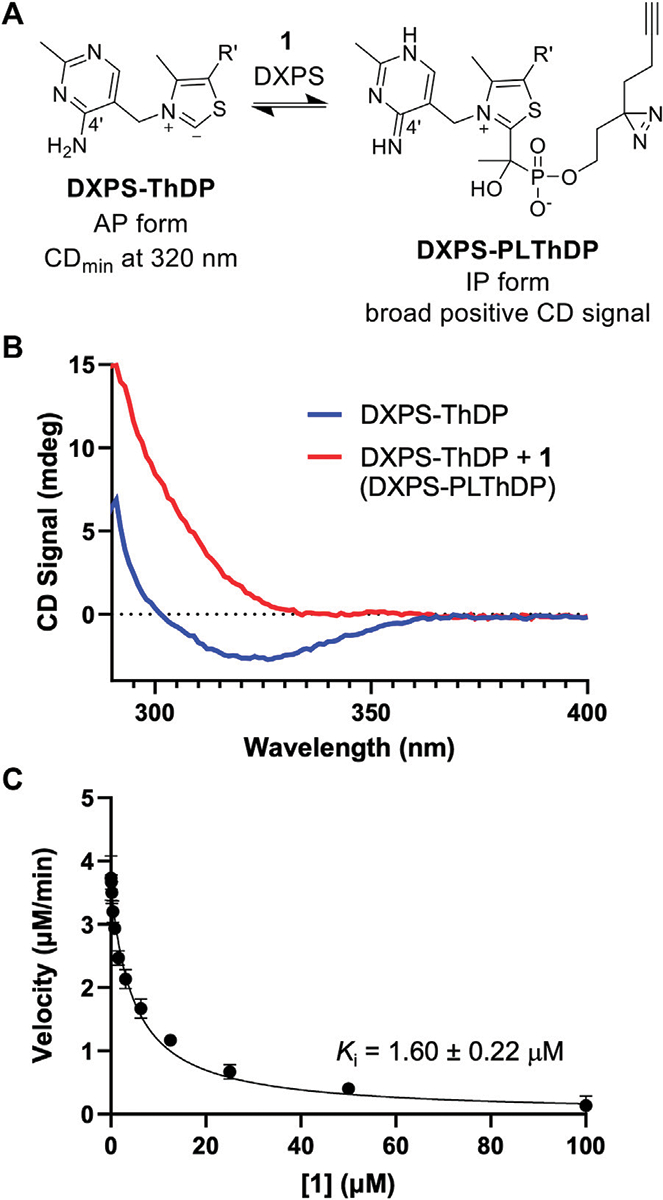
ABP **1** inhibits DXPS via PLThDP formation. **(A)** Reaction scheme for formation of PLThDP from **1** on DXPS, showing the cofactor in its AP and IP forms. **(B)** Representative CD traces showing the 4′aminopyrimidine (AP) form of ThDP on *Ec*DXPS (blue line) and PLThDP formation (red) upon addition of **1**. CD traces shown are the average of two scans. Experiments were performed in duplicate at 25°C with 50 μM **1** and 30 μM *Ec*DXPS. Replicate data shown in Supplementary Figure S3. **(C)** Morrison curve showing inhibition of DXPS activity by **1**. Error bars represent standard deviation determined from three replicates.

The phosphonolactylThDP (PLThDP) adduct formed via reaction of an alkylAP with ThDP can be detected by circular dichroism (CD) on pyruvate decarboxylase enzymes, including DXPS (Jordan et al., 2003; Nemeria et al., 2009, 2010; Heflin, 2015; Zhou et al., 2017; Coco et al., 2024). ThDP bound to DXPS exists in the 4′-aminopyrimidine (AP) form (Figure 4A) with a characteristic negative CD signal at 320 nm (Figure 4B, blue line) (Patel et al., 2012). Formation of a stable PLThDP adduct is characterized by disappearance of the negative CD signal and formation of a broad positive CD signal corresponding to the 1′,4′-iminopyrimidine (IP) form of the new PLThDP adduct (Figure 4A) (Zhou et al., 2017). As expected, formation of a broad positive CD signal was observed upon addition of **1** (50 μM) to *E. coli* DXPS (*Ec*DXPS, 30 μM) in the presence of ThDP (200 μM), supporting active site engagement of **1** with DXPS and formation of the PLThDP adduct (Figure 4B, red line).

Using the DXPS-IspC coupled assay (Coco et al., 2024) to measure initial velocity of DXP formation (Supplementary Figure S2), we assessed the ability of ABP **1** to inhibit DXPS-catalyzed DXP formation. Consistent with the observed formation of PLThDP, ABP **1** inhibits *Ec*DXPS with a *K*_i_ of 1.60 ± 0.22 μM (Figure 4C), comparable to the observed low micromolar potencies of other first-generation alkylAPs (Smith et al., 2014; Sanders et al., 2017). To rule out inhibition of the coupling system, ABP **1** was assessed as an inhibitor of IspC and found to be inactive up to 100 μM (Supplementary Figure S2B). To confirm that the potency of **1** is not due to UV-induced crosslinking during the DXPS-IspC coupled assay, the *K*_i_ of **1** was determined by measuring initial velocities of DXP formation after exposing mixtures of **1** and DXPS to 340 nm light for 5 min (Supplementary Figure S2C). A comparable *K*_i_ of 2.60 ± 0.47 μM was determined, indicating **1** is stable under conditions of the coupled assay.

### 2.3 ABP 1 labels *E. coli* DXPS in a dose-dependent manner

**FIGURE 5:**
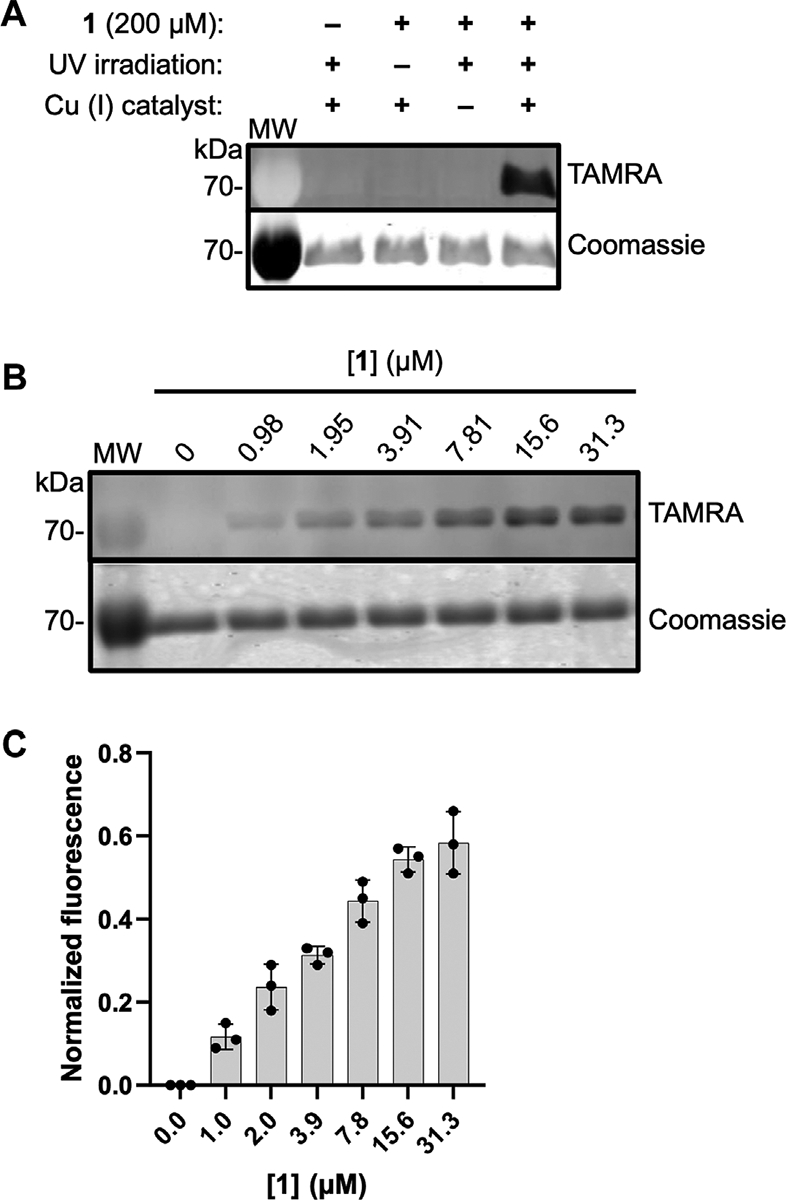
ABP 1 labels *Ec*DXPS in a dose-dependent manner. **(A)** Labeling of DXPS (3 μM) by **1** (200 μM) requires the presence of **1**, UV irradiation (3 min, 365 nm) and Cu (I) catalyst. Labeling experiments were performed in triplicate; experimental replicates as full gel images are shown in Supplementary Figure S4. **(B)** Representative in-gel fluorescence showing dose-dependent labeling of *Ec*DXPS at low micromolar concentrations of **1**; replicates as full gel images are shown in Supplementary Figure S5. **(C)** Normalized fluorescence quantified from SDS-PAGE gels (*n* = 3) showing EcDXPS (3 μM) is fully labeled at 31.3 μM **1** (samples were irradiated with 365 nm light for 3 min at 4°C). Error bars represent standard deviation. MW = protein molecular weight marker (kDa).

To evaluate the ability of **1** to label active DXPS, we carried out the workflow shown in Figure 2 in which *Ec*DXPS and **1** were incubated on ice for 10 min, then irradiated at 365 nm (180 W) for 3 min at 4°C. Crosslinked DXPS was then denatured, treated with tetramethylrhodamine (TAMRA)-azide under Cu (I)-catalyzed azide-alkyne cycloaddition (CuAAC) conditions to install the fluorophore, and evaluated by SDS-PAGE. Indeed, labeling of DXPS (3 μM) was observed in the presence of 200 μM **1** and depended upon photochemical activation of the diazirine as well as the CuAAC reaction to incorporate TAMRA, as evidenced by a lack of labeling in the absence of irradiation or Cu (I) catalyst (Figure 5A). Labeling of DXPS with **1** shows dose-dependence (Figures 5B, C), with saturation under these conditions evident at 31.3 μM **1**.

### 2.4 Labeling by 1 depends upon DXPS activity

**FIGURE 6:**
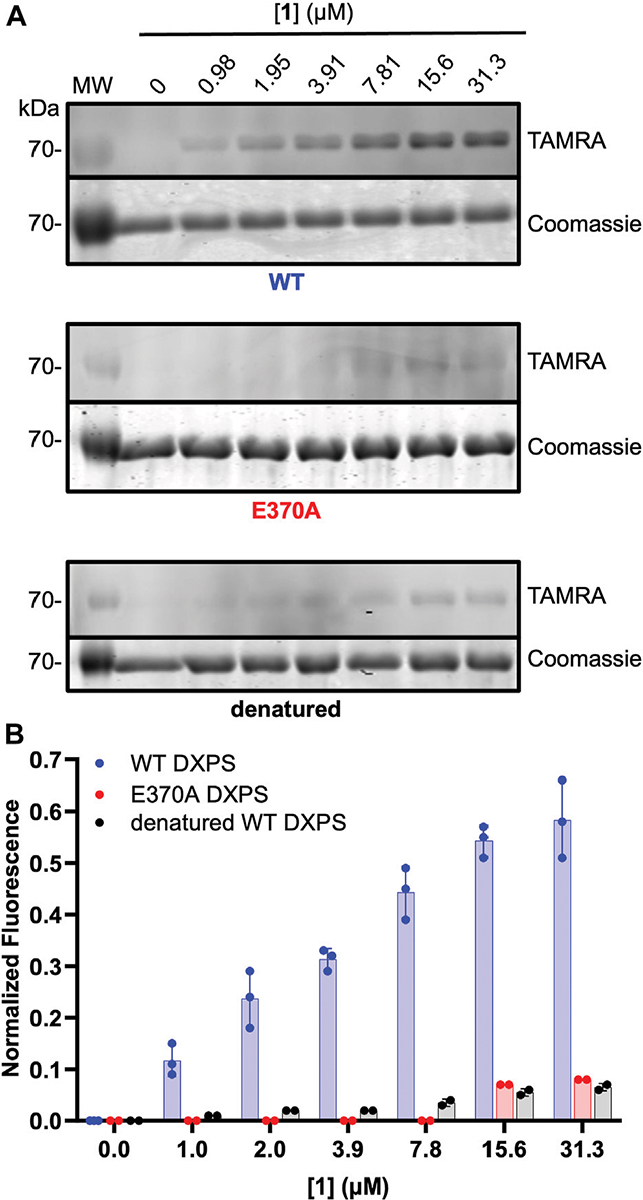
Diminished labeling of catalytically impaired *Ec*E370A DXPS and denatured wild-type DXPS by **1**. **(A)** Representative in-gel fluorescence experiments conducted with wild-type (WT) DXPS (top, data from Figure 5 included for reference (full gel images for Figure 5 data are shown in Supplementary Figure S5), EcE370ADXPS (middle), and denatured wild-type DXPS (bottom) after exposure to labeling conditions with **1**. Gel images were prepared and quantified using ImageJ. MW = protein molecular weight marker. Experimental replicates as full gel images are shown in Supplementary Figure S8. **(B)** Quantified normalized fluorescence (experimental) from in-gel fluorescence and Coomassie gel images. Error bars represent as standard deviation.

ABP **1** is designed to bind within the DXPS active site and undergo reaction with ThDP to form the PLThDP adduct; thus, efficient labeling of DXPS by **1** should be dependent upon the ability of DXPS to activate ThDP to the reactive ylide. To demonstrate this, we assessed the ability of **1** to label denatured wild-type DXPS as well as a catalytically impaired DXPS variant (*Ec*E370A DXPS) (Brammer, 2013; Querol-Audí et al., 2014). While secondary structure and stability of *Ec*E370A DXPS are similar to wild type (Supplementary Figures S6, S7), this variant lacks the conserved glutamate within hydrogen bonding distance of the cofactor N1’, required for activation of ThDP to the reactive ylide during catalysis (Muller et al., 1993; Wikner et al., 1994; Schellenberger, 1998; Schneider and Lindqvist, 1998; Berthold et al., 2005; Jordan and Nemeria, 2005; Xiang et al., 2007; Querol-Audí et al., 2014; White et al., 2016). As expected, labeling of *Ec*E370A DXPS was significantly diminished at concentrations of **1** that fully label active wild-type DXPS (>15.6 μM, Figure 6). Likewise, diminished labeling of denatured *Ec*DXPS was observed relative to active wild-type DXPS. Weak labeling could be detected at [**1**] > 7.81 μM, indicating low-level non-specific interactions between the probe and inactive DXPS. At [**1**] > 31.3 μM, more pronounced non-specific labeling is observed (Supplementary Figure S8). Together, these results indicate that reversible binding to the DXPS active site alone is insufficient for productive labeling by **1**, and conversion to the PLThDP adduct via reaction of **1** with ThDP is required.

### 2.5 DXPS inhibitors compete with 1

**FIGURE 7:**
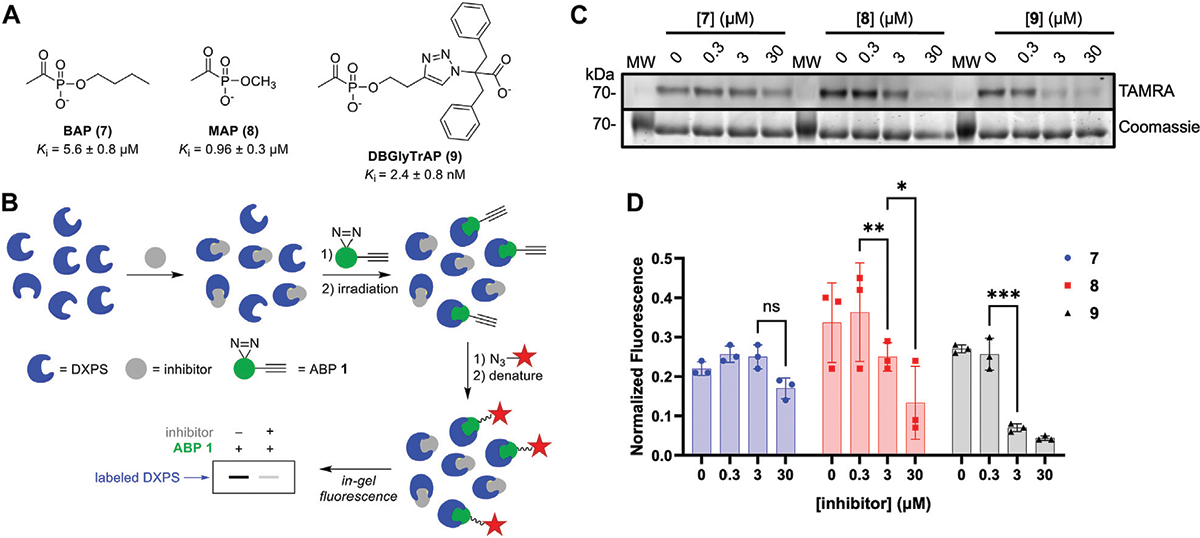
DXPS inhibitors block labeling by **1**. **(A)** Structures and potencies of alkylAP-based DXPS inhibitors. **(B)** Workflow for competitive labeling of DXPS activity. **(C)** Representative in-gel fluorescence (TAMRA) of DXPS (3 μM) labeling by **1** (50 μM) in the presence of **7**, **8** or **9** at varying concentrations. **(D)** Quantified normalized fluorescence was quantified in ImageJ. Error bars represent standard deviation of three replicates. *p < 0.05, **p < 0.01, ***p < 0.001, ns = not significant. Experiment replicates as full gel images are shown in Supplementary Figure S9.

To gain additional evidence that ABP **1** engages the DXPS active site, we conducted competition experiments using previously studied pyruvate-competitive alkylAP-based inhibitors known to act via PLThDP formation on DXPS. Three inhibitors with varying potencies were selected (Figure 7A), including butylacetylphosphonate (BAP, **7**), methylacetylphosphonate (MAP, **8**), and dibenzylglycine triazole acetylphosphonate (DBGlyTrAP, **9**) (Smith, Vierling, and Meyers, 2012; Sanders et al., 2017; Coco et al., 2024). BAP (**7**) and MAP (**8**) are first-generation alkylAPs that display low micromolar and submicromolar potencies, respectively, against DXPS enzymes. DBGlyTrAP (**9**) is a recently-discovered time-dependent bisubstrate analog inhibitor displaying low nanomolar potency against *Ec*DXPS (Coco et al., 2024). *Ec*DXPS (3 μM) was incubated with each inhibitor for 10 min prior to the addition of **1** (Figure 7B). Following a 10 min incubation with **1**, mixtures were irradiated for 3 min at 4°C, subjected to CuAAC reaction conditions to install the TAMRA fluorophore, and analyzed by SDS-PAGE, as described above. As expected, the concentration of inhibitor required to block DXPS labeling by **1** decreases with increasing inhibitor potency (Figures 7C, D); BAP (**7**) is unable to compete effectively with **1** up to 30 μM, whereas MAP (**8**) and DBGlyTrAP (**9**) block labeling by **1** in a dose-dependent manner consistent with their relative potencies. These results offer further strong evidence that ABP **1** is acting at the DXPS active site, and demonstrate the utility of **1** for identifying and characterizing inhibitor potency in DXPS inhibitor development.

### 2.6 ABP 1 displays selectivity for DXPS

**FIGURE 8:**
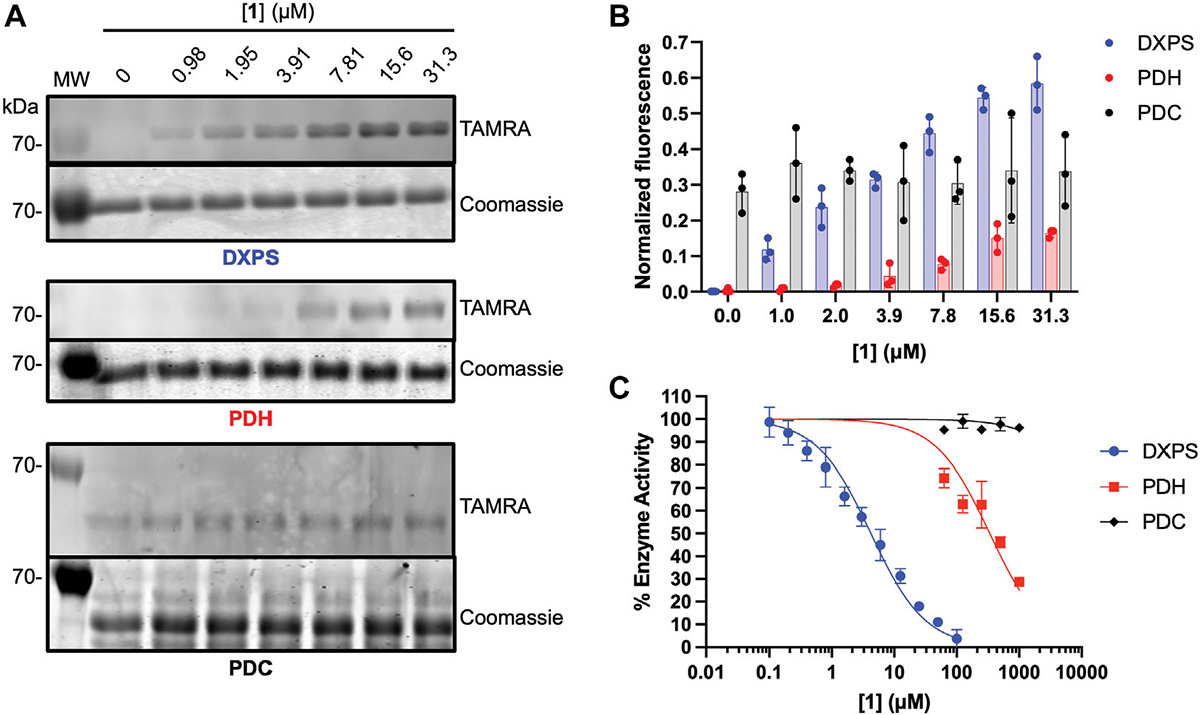
Assessment of off-target effects of **1** on ThDP-dependent pyruvate decarboxylases PDH and PDC. **(A)** Representative in-gel fluorescence analysis (TAMRA) of PDH (3 μM) and PDC (3 μM) labeling by **1**. Data for DXPS labeling from Figure 5 are included for reference (full gel images for Figure 5 data are shown in Supplementary Figure S5). **(B)** Quantification of normalized fluorescence using ImageJ. Error bars represent standard deviation from three replicates. **(C)** Inhibitory activity of **1** against *E. coli* DXPS, porcine PDH and *S. cerevisiae* PDC. Data for DXPS inhibition by ABP **1** from Figure 4C are included here, and presented as % DXPS activity, for comparison. Kinetic experiments were performed in triplicate. Initial velocities used to calculate % enzyme activity are summarized in Supplementary Table S1. Pyruvate dehydrogenase (PDH, porcine heart); pyruvate decarboxylase (PDC, *Saccharomyces cerevisiae*). Labeling experiments were performed in triplicate. Experiment replicates as full gel images are shown in Supplementary Figure S10.

As noted, alkylAPs bearing sterically demanding phosphonate ester substituents have the potential to selectively target the large active site of DXPS (Smith, Vierling, and Meyers, 2012; Morris et al., 2013; Sanders et al., 2017). To gain preliminary insights into the selectivity of **1** for DXPS, labeling experiments were performed on porcine pyruvate dehydrogenase (PDH) and *Saccharomyces cerevisiae* pyruvate decarboxylase (PDC), ThDP-dependent enzymes that also catalyze pyruvate decarboxylation. Weak labeling of PDH by **1** relative to DXPS was observed (Figures 8A, B). In agreement with this, **1** displayed weak inhibitory activity against PDH (Figure 8C; Supplementary Table S1). Interestingly, PDC appeared to have intrinsic fluorescence in the absence of **1** (Figures 8A, B). In contrast to PDH, increasing the concentration of **1** in labeling experiments did not lead to an increase in fluorescent labeling of PDC, nor was there evidence of PDC inhibition by **1** up to 1 mM (Figure 8C; Supplementary Table S1). Taken together, these results indicate **1** displays selectivity for DXPS over other ThDP-dependent pyruvate decarboxylase enzymes.

In addition, several other proteins unrelated to ThDP-dependent enzymes were subjected to labeling conditions for preliminary assessments of non-specific labeling by **1**. These included reductoisomerase IspC, the coupling enzyme used to measure DXP forming activity of DXPS (Supplementary Figure S2), glyceraldehyde 3-phosphate dehydrogenase (GADPH), alcohol dehydrogenase (ADH) and bovine serum albumin (BSA). In all cases, negligible labeling was observed up to 31.3 μM **1** (Supplementary Figure S11), conditions under which purified *Ec*DXPS is fully labeled (Figure 5). The absence of IspC labeling by **1** is also consistent with the lack of inhibitory activity of **1** against IspC (Supplementary Figure S2). These results suggest minimal non-specific interactions of **1** under these conditions.

### 2.7 ABP 1 labels DXPS in complex bacterial lysate

**FIGURE 9:**
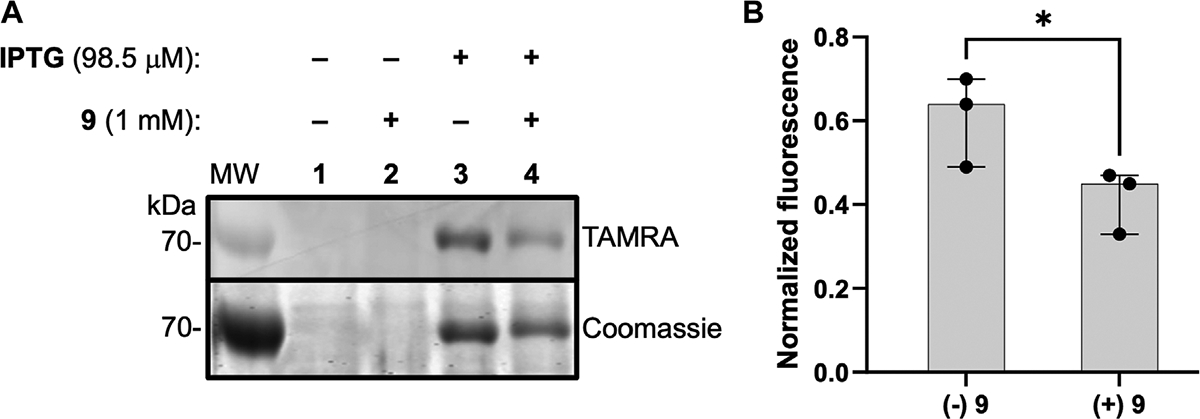
ABP 1 labels active DXPS in bacterial lysate. **(A)** DXPS labeling by **1** (1 mM) observed in bacterial lysate from DXPS-overexpressing *E. coli* BL21 (DE3) cells (induced with IPTG, lane 3). No labeling is observed in the absence of IPTG induction (lanes 1 and 2). Incubation of **9** (1 mM) with lysate from IPTG-induced cells blocks labeling by **1** to some extent (lane 4). Replicate data as full gel images shown in Supplementary Figure S12. **(B)** Quantification of in-gel fluorescence of labeled DXPS in the presence or absence of **9** (1 mM), normalized to Coomassie-stained DXPS, shows a statistically significant reduction in DXPS labeling by **1** in the present of **9**; *n* = 3, *p < 0.05.

As a first step to evaluate **1** as a probe of DXPS activity in a complex proteome, we assessed labeling of DXPS by **1** in lysate from DXPS-overexpressing *E. coli*. Bacterial lysates were prepared from *E. coli* BL21 (DE3) cells harboring the *dxs*-pET37b expression construct for inducible expression of DXPS, the strain used for production and purification of recombinant DXPS (Brammer and Meyers, 2009). In lysate prepared from isopropyl β-D-1-thiogalactopyranoside (IPTG)-induced cultures, DXPS overexpression was observed, and labeling of DXPS by **1** is evident (Figure 9A, lane 3), compared to a lack of overexpression and DXPS labeling in lysate prepared from uninduced cultures (Figure 9A, lane 1). Further, incubation with **9** led to a reduction in labeling by **1** (Figure 9A, lane 4; Figure 9B). Taken together, these results suggest a potential utility of **1** to probe DXPS activity in complex proteomes and as a tool for DXPS inhibitor discovery and development.

## 3 Discussion

Previous studies of DXPS have shown how DXPS activity and mechanism are distinct within the ThDP-dependent enzyme family, providing avenues for selective inhibition and offering a molecular basis for potential multifunctionality of this enzyme. Our discovery of a DXPS function in adaptation, and finding that a pathogen can be uniquely sensitized to DXPS inhibition in different environments, suggest DXPS activity and/or function may be distinct in different contexts of infection. At present, this is minimally understood, and tools to probe the various ways pathogens exploit DXPS activity during infection are lacking.

This study sought to take initial steps toward development of activity-based probes that can be used to study DXPS biology and aid in drug discovery efforts. AlkylAP-based inhibitors developed previously in our lab proved an excellent starting point for the design of ABPs capable of detecting DXPS activity. Given that alkyAPs act by reversible PLThDP formation on DXPS, we designed a probe that incorporates a commonly-used alkyne bearing a diazirine crosslinking group for irreversible labeling. ABP **1**, synthesized in 5 steps from readily available starting materials, was found to act as an inhibitor of DXPS via formation of the corresponding PLThDP adduct, as expected for an alkylAP. Notably, dose-dependent labeling by **1** was observed only for active DXPS at concentrations of **1** up to 31.3 μM; the inactive *Ec*E370A variant was inaccessible to labeling under these conditions, despite having similar secondary structure to wild-type DXPS. This is consistent with prior results showing overexpression of the *Ec*E370A variant does not suppress cellular activity of **7** (Sanders et al., 2017), and indicates conversion of **1** to the PLThDP adduct is necessary to achieve the affinity required for efficient crosslinking. Labeling of active DXPS was partially or fully blocked by alkylAPs displaying enzyme inhibitory activity in the low micromolar to low nanomolar range; concentration-dependent reduction in labeling correlated with inhibitor potencies. Together these results provided additional strong evidence that ABP **1** engages the DXPS active site and effectively reports on DXPS activity. Additionally, the observed potent competition by **9** supports the use of ABP **1** as a tool for inhibitor discovery and suggests the bisubstrate scaffold as a promising starting point for second-generation ABPs for DXPS.

Weak labeling and inhibition of ThDP-dependent pyruvate dehydrogenase by **1** was observed, consistent with our finding that increasing alkyl chain length beyond four carbons modestly increases alkylAP potency against PDH (Sanders et al., 2017). In contrast, **1** did not efficiently label or inhibit the related pyruvate decarboxylase or other mechanistically unrelated enzymes. This suggested some level of specificity of ABP **1** for DXPS, which is expected to help mitigate inefficient DXPS labeling in more complex environments. Importantly, preliminary evaluation of **1** as a probe of DXPS activity in a complex proteome showed that DXPS could be labeled in bacterial lysate from DXPS-overexpressing *E. coli*, and the most potent alkylAP (DBGlyTrAP **9**) was capable of blocking DXPS labeling to some extent in this condition. Inhibitor **9** did not fully outcompete **1** in this condition, despite its 600-fold higher potency against DXPS relative to **1**. This points to non-specific labeling in the presence of 1 mM **1**, which is plausible based on the non-specific labeling of purified protein observed in the presence of [**1**] > 31.3 μM (Supplementary Figure S8). Nevertheless, these results indicate DXPS is active in this condition and susceptible to inhibition by **9**. Further optimization of lysate preparation and ABP concentration is required to evaluate the ability of **1** to detect native DXPS activity in complex lysates generated under varying growth conditions. The altered alkylAP antimicrobial activity observed in different growth conditions (Sanders et al., 2017, 2018; E. C. Chen and Freel Meyers, 2023) could reflect changes in inhibitor permeability and access to DXPS. These differences could also signify remodeling of the metabolic or regulatory networks controlling DXPS activity and/or DXPS-inhibitor affinity. Overall, the results of this work represent a promising step toward using alkylAP-based ABPs to interrogate DXPS biology and to evaluate new DXPS inhibitors in different contexts.

## 4 Materials and methods

### 4.1 General

Chemicals were purchased from Millipore Sigma (Sigma-Aldrich) and used as received, unless otherwise stated. TAMRA-azide (CCT-AZ109) was purchased from Vector Laboratories (Click Chemistry Tools). *E. coli* DXPS and IspC were overexpressed, purified using an AKTA-GO fast protein liquid-chromatography (FPLC) system, and characterized as previously reported (Coco et al., 2024). Bovine serum album (BSA), glyceraldehyde 3-phosphate (GAPDH, rabbit muscle), alcohol dehydrogenase (ADH, *S. cerevisiae*), pyruvate dehydrogenase (PDH, porcine heart) and pyruvate decarboxylase (PDC, *S. cerevisiae*) were purchased from Millipore Sigma (Sigma-Aldrich). The *E. coli* BL21 (DE3) cell line harboring the *dxs*-pET37b plasmid (Brammer and Meyers, 2009), for inducible DXPS overexpression, was used in experiments to investigate labeling of DXPS by **1** in complex lysate. DXPS inhibitors **7**–**9** were prepared as previously described (Smith, Vierling, and Meyers, 2012; Sanders et al., 2017; Coco et al., 2024). A BioTek Epoch 2 microplate reader was used at 25°C for aerobic spectrophotometric analyses. A Li-COR Odyssey CLx was used for imaging Coomassie-stained SDS-PAGE gels. Fluorescent gels were scanned using an Amersham Typhoon (Cytiva) biomolecular imager (excitation 554 nm; emission 566 nm). Circular dichroism (CD) experiments were performed on an Applied Photophysics Chirascan V100 CD spectrometer (Surrey, United Kingdom). A Spectroline Model FC-100 lamp with longwave ultraviolet light (365 nm, 180 W) was used for crosslinking studies. Protostain Blue (colloidal Coomassie Blue G-250) was used to stain proteins in SDS-PAGE analysis.

### 4.2 Circular dichroism (CD) to detect PLThDP formation from 1 on DXPS

DXPS (30 μM) was diluted to a final volume of 1.5 mL in enzyme buffer (1 mM MgCl_2_, 100 mM NaCl, 200 μM ThDP, 50 mM HEPES pH 8) on ice in a conical tube. Immediately prior to CD experiments, protein solutions were equilibrated at 25°C for 10 min. Sample was then transferred from the conical tube to a 1 cm quartz cuvette and a CD scan was recorded at 25°C from 280–400 nm with a 2 nm step and 0.5 s averaging time. ABP **1** was added to a final concentration of 50 μM (3 μL of 25 mM stock), the cuvette was inverted gently 3 times to mix, and a second CD scan was recorded on the mixture at 25°C using the same parameters as the initial scan. Experiments were performed in duplicate.

### 4.3 Determination of *K*_i_ of 1 with wild-type DXPS

The DXPS-IspC coupled assay (Coco et al., 2024) was used to measure the rate of DXPS-catalyzed DXP formation in the presence or absence of **1**. DXPS (100 nM) and IspC (2 μM) were preincubated with **1** (0–100 μM) in buffer (2 mM MgCl_2_, 5 mM NaCl, 1 mM ThDP, 100 mM HEPES pH 8, 200 μM NADPH) at 25°C for 10 min. Enzyme reactions were initiated by the addition of substrates (50 μM pyruvate and 500 μM D-GAP). The change in absorbance of NADPH at 340 nm was monitored at 25°C and used to calculate the initial velocity of DXP formation. Initial velocities were plotted as a function of [**1**]. Data were fit to the Morrison equation (Morrison, 1969) (Eq. 1) to calculate *K*_i_. Non-linear regression analysis was performed using GraphPad Prism version 10. Error bars represent standard deviation. Standard deviation was calculated from three replicate *K*_i_ determinations (*K*_i_ ± SD).


(1) $$\frac{v_{i}}{v_{0}}=1-\frac{\left({\left[E\right]}_{T}+{\left[I\right]}_{T}+K_{i}\right)-\sqrt{{\left({\left[E\right]}_{T}+{\left[I\right]}_{T}+K_{i}\right)}^{2}-4{\left[E\right]}_{T}{\left[I\right]}_{T}}}{2{\left[E\right]}_{T}}$$

### 4.4 General protocol for DXPS labeling by 1

ABP **1** was incubated with purified protein (3 μM) in buffer (2 mM MgCl_2_, 5 mM NaCl, 1 mM ThDP, 100 mM HEPES pH 8) for 10 min on ice. The solutions were then irradiated (365 nm, 180 W) for 3 min in a cold room (4°C). The mixture (10 μL) was subjected to denaturing conditions by addition to 10% sodium dodecyl-sulfate (SDS, 10 μL) following by vortexing and heating (5 min at 95°C). Solutions were cooled to ambient temperature, and a 7.5× CuAAC reaction stock (4 μL, prepared immediately prior to addition) was added to achieve final concentrations of 1 mM tris ((1-benzyl-4-triazolyl)methyl)amine (TBTA), 10 mM CuSO_4_, and 10 mM tris(2-carboxyethyl)phosphine (TCEP). TAMRA-azide (6 μL of 5 mM stock in DMSO) was then added to initiate the reaction. The reaction mixture was incubated for 1 h at ambient temperature, covered by foil. SDS-PAGE loading dye (10 μL of 4× solution containing 200 μM Tris-HCl, 400 μM dithiothreitol, 277 mM SDS, 6 mM bromophenol blue, and 4.3 M glycerol) was added, the mixture was vortexed, heated (5 min at 95°C), and a 15 μL aliquot was analyzed by SDS-PAGE (10% acrylamide). Gels were first scanned to detect in-gel fluorescence and then stained with Protostain blue (colloidal Coomassie Blue G-250) to visualize total protein. Gel images were generated and quantified using ImageJ. For experiments in which labeled enzyme was quantified, pixel densities of fluorescently labeled protein were determined using ImageJ. Briefly, the HiLo threshold command in ImageJ was employed, and contrast was adjusted such that no part of the image exceeded the maximum threshold. Vertical rectangular boxes were drawn to encompass each protein band, and pixel density across the band within the box was plotted. Pixel density was determined by integrating the plotted signal. Fluorescently labeled protein was then normalized to Coomassie-stained protein (Eq. 2).


(2) $$\text{Normalized}\;\text{fluorescence}=\frac{\text{pixel}\;\text{densit}y_{\text{TAMRA}\;\text{gel}\;\text{band}}}{\text{pixel}\;\text{densit}{\text{y}}_{\text{Coomassie}\;\text{gel}\;\text{band}}}$$

#### 4.4.1 Labeling control experiments

The general protocol for labeling by **1** was employed with minor adjustments. All samples contained 200 μM **1**, with the exception of the (−) probe control. All samples were subjected to UV irradiation with the exception of the (−) UV control reaction which was protected from light under foil. All samples contained Cu (I) (from CuSO_4_ under reducing conditions), with the exception of the (−) Cu (I) control for the attachment of the TAMRA-azide. The positive control contained all components and was carried through all steps. Experiments were performed in triplicate.

#### 4.4.2 Dose-dependent labeling of DXPS by 1

The general protocol for labeling by **1** was employed. DXPS was maintained at 3 μM in buffer (2 mM MgCl_2_, 5 mM NaCl, 1 mM ThDP, 100 mM HEPES pH 8). The DXPS-containing solution (90 μL) was mixed with **1** (10 μL of 10× solutions to achieve final concentrations of **1** between 0–31.3 μM). Mixtures were incubated for 10 min on ice prior to irradiation. Experiments were performed in triplicate and pixel density of fluorescently labeled protein was quantified by ImageJ and normalized to Coomassie-stained DXPS as described above.

#### 4.4.3 Labeling of wild-type DXPS, heat-inactivated, and E370A DXPS

The general protocol for labeling by **1** was employed. To denature wild-type DXPS, a 50 μL of DXPS (113 μM stock) was vortexed for 20 min at ambient temperature, followed by incubation at 75°C for 30 min, after which insoluble aggregate was visible. Denatured DXPS was then diluted 2-fold with 10% SDS to solubilize the aggregate, and left overnight at ambient temperature. E370A generated by previously reported methods was found to retain only 0.12% DXP forming activity relative to wild-type DXPS (Brammer, 2013; Querol-Audí et al., 2014). Labeling experiments were conducted as described above. The *Ec*E370A variant and denatured wild-type DXPS were subjected to labeling by **1** at varying concentrations (0–125 μM) as described above and compared to the dose-dependent labeling of wild-type DXPS above. Labeling of E370A and denatured wild-type DXPS were conducted in duplicate. Pixel density of fluorescently labeled protein was quantified by ImageJ and normalized to Coomassie-stained DXPS as described above.

#### 4.4.4 Competition with alkylAPs

AlkylAP inhibitor (10 μL of 10× solution of **7**, **8** or **9**) was added to a solution of DXPS in buffer (80 μL, 2 mM MgCl_2_, 5 mM NaCl, 1 mM ThDP, 100 mM HEPES pH 8) to achieve final concentrations of 0, 0.3, 3, and 30 μM inhibitor. The mixture was incubated for 10 min at ambient temperature. Then, the general protocol for labeling by **1** was employed. Briefly, **1** (10 μL of 10× solution) was added to the DXPS-inhibitor mixture to a final concentration of 50 μM, and the mixture was incubated for an additional 10 min at ambient temperature, under foil. Samples were then irradiated (365 nm, 3 min, 4°C) and analyzed by SDS-PAGE as described in the general protocol for labeling **1** above. Experiments were performed in triplicate and pixel density of fluorescently labeled protein was quantified by ImageJ and normalized to Coomassie-stained DXPS as described above.

#### 4.4.5 Labeling of PDH and PDC by 1

The general protocol for labeling by **1** was employed with the following adjustments. PDH (porcine heart, 3 μM) was subjected to labeling by **1** (0–31.3 μM) in buffer containing 2 mM MgCl_2_, 1 mM ThDP, 0.3 mM TCEP, and 100 mM HEPES pH 8. PDC (*S. cerevisiae*, 3 μM) was subjected to labeling by **1** (0–31.3 μM) in buffer containing 100 mM 2-(*N*-morpholino)ethanesulfonic acid (MES) pH 6, 2 mM MgCl_2_, and 1 mM ThDP. Experiments were performed in triplicate and pixel density of fluorescently labeled protein was quantified by ImageJ and normalized to Coomassie-stained DXPS as described above.

#### 4.4.6 Labeling of BSA, GAPDH, ADH and IspC by 1

The general protocol for labeling by **1** was employed with the following adjustments. Proteins were subjected to labeling by **1** over the concentration range 0–31.3 μM. Labeling of BSA was conducted in buffer containing 2 mM MgCl_2_, 5 mM NaCl, 1 mM ThDP, and 100 mM HEPES pH 8. Labeling of GAPDH was conducted in buffer containing 1 mM MgCl_2_, 100 mM NaCl, and 30 mM GlyGly pH 8. Labeling of ADH was conducted in buffer containing 2 mM MgCl_2_, 100 mM NaCl, 1 mM ThDP, and 100 mM HEPES pH 8. Labeling of IspC was conducted in buffer containing 2 mM MgCl_2_ and 100 mM HEPES pH 8. Experiments were performed in duplicate. Pixel density of fluorescently labeled protein was quantified by ImageJ and normalized to Coomassie-stained DXPS as described above.

### 4.5 Determination of inhibitory activity of 1 against PDH and PDC

PDH (0.01 U/mL) was preincubated with **1** (0–1,000 μM) for 10 min at 25°C in buffer containing 100 mM HEPES pH 8, 2 mM MgCl_2_, 5 mM L-cysteine, 1 mM ThDP, 0.3 mM TCEP, 2.5 mM NAD^+^, and 100 μM Coenzyme A. The enzyme reaction was initiated by addition of pyruvate (60 μM) at 25°C. Initial velocity was determined by measuring NADH formation at 340 nm, and normalized to initial velocity in the absence of **1** to calculate % activity which was plotted as a function of [**1**] using GraphPad Prism. Error bars represent standard deviation of the fit from three replicates.

Inhibitory activity of **1** against PDC was determined as described above with the following modifications. PDC (0.05 U/mL) activity was assayed in buffer containing 100 mM 2-(*N*-morpholino) ethanesulfonic acid (MES) pH 6, 5 mM MgCl_2_, 5 mM ThDP, 0.17 mM NADH, and 16 U/mL alcohol dehydrogenase as the coupling enzyme to detect formation of acetaldehyde product. The enzyme reaction was initiated by the addition of pyruvate (1 mM). Initial velocity was determined by measuring NADH depletion at 340 nm.

### 4.6 Detection of DXPS in complex bacterial lysate

#### 4.6.1 Preparation of lysate from DXPS-overexpressing BL21 (DE3) *E. coli*

Sterile lysogeny broth (LB) (3 mL containing 50 μg/mL kanamycin) was inoculated with BL21 (DE3) *E. coli* harboring the DXPS-overexpression plasmid *dxs*-pET37b, from a glycerol stock. The culture was incubated overnight at 37°C with shaking. The saturated overnight culture (300 μL) was added to two separate culture tubes each containing fresh LB broth (20 mL containing 50 μg/mL kanamycin) and the resulting culture was incubated at 37°C with shaking until an OD_600_ of ~0.7 was reached. To one culture, isopropyl β-D-1-thiogalactopyranoside (98.5 μM IPTG, 2 μL of 1 M stock in water) was added to induce DXPS expression (induced). To the second culture, water (2 μL) was added (uninduced). Both cultures were incubated with shaking for an additional 4 h at 37°C, then centrifuged at 4,000 × g for 10 min at 4°C. The culture medium was decanted, and the remaining cell pellet was stored at −80°C overnight. The following day, the pellet was thawed and washed to remove remaining LB medium by resuspension in 5 mL lysis buffer containing 400 mM NaCl, 50 mM Tris pH 8, 20 mM MgCl_2_, 10% v/v glycerol, 1 mM phenylmethylsulfonyl fluoride (PMSF), 6 μL/40 mL DNase, and 1× protease inhibitor cocktail (PIC, Millipore Sigma P8849); resulting suspensions were centrifuged (4,000 × g, 10 min at 4°C), and the supernatant was decanted. The resulting cell pellet was resuspended in 2 mL lysis buffer. Cells were lysed by sonication and the resulting crude lysate was centrifuged to remove insoluble material (4,000 × g at 4°C). The supernatant was collected and used for labeling experiments. Bacterial lysate was prepared by this protocol in triplicate, from 3 separate cultures grown from the glycerol stock of *E. coli* BL21 (DE3) cells harboring *dxs*-pET37b.

#### 4.6.2 Labeling of DXPS by 1 in lysate

Lysate (80 μL) from IPTG-induced or uninduced cells, prepared as described above, was transferred to a 96-well plate (2 wells containing lysate from induced cells, and 2 wells containing lysate from uninduced cells). Water (10 μL) or **9** (10 μL of 10 mM stock in water) was added to lysate, and the mixtures were incubated for 20 min at 25°C. ABP **1** (10 μL of a 10 mM stock in water) was then added (both **9** and **1** present at a final concentration of 1 mM), and the mixture was incubated for an additional 20 min at 25°C, under foil. Mixtures were irradiated (365 nm) for 3 min at 4°C. An aliquot of the crosslinked mixture (10 μL) was added to 10% SDS (10 μL). Samples were vortexed and heated (5 min at 95°C). A 7.5× CuAAC reaction stock (1 mM TBTA, 10 mM CuSO_4_, and 10 mM TCEP) was prepared and 4 μL was added to each sample followed by addition of TAMRA-azide (6 μL of a 5 mM stock in DMSO). The CuAAC reaction proceeded for 1 h at ambient temperature under foil. Loading dye (10 μL of a 4× stock) was added to each CuAAC reaction mixture, and 15 μL of the resulting mixture was analyzed by SDS-PAGE (10% acrylamide). Gels were first scanned to detect fluorescent labeling and then stained with Protostain blue (colloidal Coomassie Blue G-250) to visualize total protein. Gel images were generated using ImageJ. Experiments were performed on lysate preparation triplicates.

## Supplementary Material

Supplementary Material

## Data Availability

The original contributions presented in the study are included in the article/Supplementary Material, further inquiries can be directed to the corresponding author.

## Nomenclature

DXPS: 1-deoxy-d-xylulose 5-phosphate synthase
ThDP: thiamin diphosphate
d-GAP: d-glyceraldehyde 3-phosphate
PLP: pyridoxal phosphate
MEP: methylerythritol phosphate
UPEC: uropathogenic *E. coli*
ABP: activity-based probe
LThDP: C2α-lactylthiamin diphosphate
AlkylAP: alkyl acetylphosphonate
PLThDP: phosphonolactylthiamin diphosphate
PDH: pyruvate dehydrogenase
PDC: pyruvate decarboxylase
CD: circular dichroism
AP: 4′-aminopyrimidine tautomer of ThDP
IP: 1′,4′-iminopyrimidine tautomer of ThDP
IspC: 1-deoxy-d-xylulose 5-phosphate reductoisomerase
CuAAC: Cu(I)-catalyzed azide-alkyne cycloaddition
BAP: butyl acetylphosphonate
MAP: methyl acetylphosphonate
DBGlyTrAP: dibenzylglycine triazole acetylphosphonate
GAPDH: glyceraldehyde 3-phosphate dehydrogenase
ADH: alcohol dehydrogenase
BSA: bovine serum albumin
IPTG: isopropyl β-d-1-thiogalactopyranoside
HEPES: 4-(2-Hydroxyethyl)-1-piperazineethanesulfonic acid
NADPH: nicotinamide adenine dinucleotide phosphate
TBTA: tris ((1-benzyl-4-triazolyl)methyl)amine
TCEP: tris (2-carboxyethyl)phosphine
MES: 2-(*N*-morpholino)ethanesulfonic acid
NAD^+^: nicotinamide adenine dinucleotide
NADH: reduced form of NAD^+^

## References

[R1] Allamand A, Piechowiak T, Lièvremont D, Rohmer M, and Grosdemange-Billiard C (2023). The multifaceted MEP pathway: towards new therapeutic perspectives. Molecules 28 (3), 1403. doi:10.3390/molecules28031403 36771066 PMC9919496

[R2] Alteri CJ, Himpsl SD, Shea AE, and Mobley HLT (2019). Flexible metabolism and suppression of latent enzymes are important for Escherichia coli adaptation to diverse environments within the host. J. Bacteriol 201 (16), 001811–e219. doi:10.1128/JB.00181-19 PMC6657593 31160397

[R3] Alteri CJ, and Mobley HLT (2012). Escherichia coli physiology and metabolism dictates adaptation to diverse host microenvironments. Curr. Opin. Microbiol 15 (1), 3–9. doi:10.1016/j.mib.2011.12.004 22204808 PMC3265668

[R4] Alteri CJ, and Mobley HLT (2015). Metabolism and fitness of urinary tract pathogens. Microbiol. Spectr 3 (3). doi:10.1128/microbiolspec.MBP-0016-2015 PMC4510461 26185076

[R5] Alteri CJ, Smith SN, and Mobley HLT (2009). Fitness of Escherichia coli during urinary tract infection requires gluconeogenesis and the TCA cycle. PLoS Pathog 5 (5), e1000448. doi:10.1371/journal.ppat.1000448 19478872 PMC2680622

[R6] Bartee D, and Freel Meyers CL (2018a). Targeting the unique mechanism of bacterial 1-Deoxy-d-Xylulose-5-Phosphate synthase. Biochemistry 57 (29), 4349–4356. doi:10.1021/acs.biochem.8b00548 29944345 PMC6057799

[R7] Bartee D, and Freel Meyers CL (2018b). Toward understanding the Chemistry and biology of 1-Deoxy-d-Xylulose 5-phosphate (DXP) synthase: a unique antimicrobial target at the heart of bacterial metabolism. Accounts Chem. Res 51 (10), 2546–2555. doi:10.1021/acs.accounts.8b00321 PMC6309272 30203647

[R8] Berthold CL, Moussatche P, Richards NGJ, and Lindqvist Y (2005). Structural basis for activation of the thiamin diphosphate-dependent enzyme oxalyl-CoA decarboxylase by adenosine diphosphate. J. Biol. Chem 280 (50), 41645–41654. doi:10.1074/jbc.m509921200 16216870

[R9] Brammer LA (2013). Toward investigating DXP synthase as a new anti-infective target (doctoral dissertation). PhD, Baltimore, MD: The Johns Hopkins University School of Medicine.

[R10] Brammer LA, and Meyers CF (2009). Revealing substrate promiscuity of 1-deoxy-D-xylulose 5-phosphate synthase. Org. Lett 11 (20), 4748–4751. doi:10.1021/ol901961q 19778006 PMC2761658

[R11] Chan CCY, and Lewis IA (2022). Role of metabolism in uropathogenic Escherichia coli. Trends Microbiol 30 (August), 1174–1204. doi:10.1016/j.tim.2022.06.003 35941063

[R12] Chen EC, and Freel Meyers CL (2023). DXP synthase function in a bacterial metabolic adaptation and implications for antibacterial strategies. Antibiotics 12 (4), 692. doi:10.3390/antibiotics12040692 37107054 PMC10135061

[R13] Chen PY-T, DeColli AA, Freel Meyers CL, and Drennan CL (2019). X-ray crystallography-based structural elucidation of enzyme-bound intermediates along the 1-Deoxy-d-Xylulose 5-phosphate synthase reaction coordinate. J. Biol. Chem 294 (33), 12405–12414. doi:10.1074/jbc.ra119.009321 31239351 PMC6699841

[R14] Coco LB, Toci EM, Chen PY-T, Drennan CL, and Freel Meyers CL (2024). Potent inhibition of E. Coli DXP synthase by a gem-diaryl bisubstrate analog. ACS Infect. Dis 10, 1312–1326. doi:10.1021/acsinfecdis.3c00734 38513073 PMC11019550

[R15] David S, Estramareix B, Fischer JC, and Therisod M (1981). 1-Deoxy-D-Threo-2-Pentulose: the precursor of the five-carbon chain of the thiazole of thiamine. J. Am. Chem. Soc 103 (24), 7341–7342. doi:10.1021/ja00414a053

[R16] DeColli AA, Nemeria NS, Majumdar A, Gerfen GJ, Jordan F, and Freel Meyers CL (2018). Oxidative decarboxylation of pyruvate by 1-Deoxy-d-Xyulose 5-phosphate synthase, a central metabolic enzyme in bacteria. J. Biol. Chem 293 (28), 10857–10869. doi:10.1074/jbc.ra118.001980 29784878 PMC6052232

[R17] DeColli AA, Zhang, Xu, Heflin KL, Jordan F, and Freel Meyers CL (2019). Active site histidines link conformational dynamics with catalysis on anti-infective target 1-Deoxy-d-Xylulose 5-phosphate synthase. Biochemistry 58 (49), 4970–4982. doi:10.1021/acs.biochem.9b00878 31724401 PMC6905430

[R18] Fuchs TM, Eisenreich W, Heesemann J, and Goebel W (2012). Metabolic adaptation of human pathogenic and related nonpathogenic bacteria to extra- and intracellular habitats. FEMS Microbiol. Rev 36 (2), 435–462. doi:10.1111/j.1574-6976.2011.00301.x 22092350

[R19] Heflin KL (2015). “Investigating the mechanism of DXP synthase catalysis,” in Freel Meyers. Doctor of philosophy Editor Caren L (Baltimore, MD: Johns Hopkins University). Available at: https://jscholarship.library.jhu.edu/handle/1774.2/60517.

[R20] Heuston S, Begley M, Gahan CGM, and Hill C (2012). Isoprenoid biosynthesis in bacterial pathogens. Microbiology 158 (Pt 6), 1389–1401. doi:10.1099/mic.0.051599-0 22466083

[R21] Hill RE, Sayer BG, and Spenser ID (1989). Biosynthesis of vitamin B6: incorporation of D-1-deoxyxylulose. J. Am. Chem. Soc 111 (5), 1916–1917. doi:10.1021/ja00187a076

[R22] Himpsl SD, Shea AE, Zora J, Stocki JA, Foreman D, Alteri CJ, (2020). The oxidative fumarase FumC is a key contributor for E. Coli fitness under iron-limitation and during UTI. PLoS Pathog 16 (2), e1008382. doi:10.1371/journal.ppat.1008382 32106241 PMC7064253

[R23] Johnston ML, and Freel Meyers CL (2021). Revealing donor substrate-dependent mechanistic control on DXPS, an enzyme in bacterial central metabolism. Biochemistry 60 (12), 929–939. doi:10.1021/acs.biochem.1c00019 33660509 PMC8015787

[R24] Johnston ML, Toci EM, DeColli AA, and Freel Meyers CL (2022). Antibacterial target DXP synthase catalyzes the cleavage of D-xylulose 5-phosphate: a study of ketose phosphate binding and ketol transfer reaction. Biochemistry 61 (17), 1810–1823. doi:10.1021/acs.biochem.2c00274 35998648 PMC9531112

[R25] Jordan F, and Nemeria NS (2005). Experimental observation of thiamin diphosphate-bound intermediates on enzymes and mechanistic information derived from these observations. Bioorg. Chem 33 (3), 190–215. doi:10.1016/j.bioorg.2005.02.001 15888311 PMC4189838

[R26] Jordan F, Nemeria NS, Zhang S, Yan Y, Arjunan P, and Furey W (2003). Dual catalytic apparatus of the thiamin diphosphate Coenzyme: acid-base via the 1’,4’-iminopyrimidine tautomer along with its electrophilic role. J. Am. Chem. Soc 125 (42), 12732–12738. doi:10.1021/ja0346126 14558820

[R27] Kluger R, and Pike DC (1977). Active site generated analogs of reactive intermediates in enzymic reactions. Potent inhibition of pyruvate dehydrogenase by a phosphonate analog of pyruvate. J. Am. Chem. Soc 99 (13), 4504–4506. doi:10.1021/ja00455a052 864125

[R28] Kluger R, and Tsui WC (1986). Reaction of the anionic acetylation agent methyl acetyl phosphate with D-3-hydroxybutyrate dehydrogenase. Biochem. Cell Biol 64 (5), 434–440. doi:10.1139/o86-061 3718711

[R29] Li Z, Hao P, Li L, Tan CYJ, Cheng X, Chen GYJ, (2013). Design and synthesis of minimalist terminal alkyne-containing diazirine photo-crosslinkers and their incorporation into kinase inhibitors for cell- and tissue-based proteome profiling. Angew. Chem. Int. Ed 52 (33), 8551–8556. doi:10.1002/anie.201300683 23754342

[R30] Morris F, Vierling R, Boucher L, Bosch J, and Freel Meyers CL (2013). DXP synthase-catalyzed C-N bond formation: nitroso substrate specificity studies guide selective inhibitor design. ChemBioChem 14 (11), 1309–1315. doi:10.1002/cbic.201300187 23824585 PMC3767973

[R31] Morrison JF (1969). Kinetics of the reversible inhibition of enzyme-catalysed reactions by tight-binding inhibitors. Biochimica Biophysica Acta (BBA) - Enzym. 185 (2), 269–286. doi:10.1016/0005-2744(69)90420-3 4980133

[R32] Muller YA, Lindqvist Y, Furey W, Schulz GE, Jordan F, and Schneider G (1993). A thiamin diphosphate binding fold revealed by comparison of the crystal structures of transketolase, pyruvate oxidase and pyruvate decarboxylase. Structure 1 (2), 95–103. doi:10.1016/0969-2126(93)90025-c 8069629

[R33] Murima P, McKinney JD, and Pethe K (2014). Targeting bacterial central metabolism for drug development. Chem. Biol 21 (11), 1423–1432. doi:10.1016/j.chembiol.2014.08.020 25442374

[R34] Nemeria NS, Arjunan P, Chandrasekhar K, Mossad M, Tittmann K, Furey W, (2010). Communication between thiamin cofactors in the Escherichia coli pyruvate dehydrogenase complex E1 component active centers. J. Biol. Chem 285 (15), 11197–11209. doi:10.1074/jbc.m109.069179 20106967 PMC2856997

[R35] Nemeria NS, Chakraborty S, Balakrishnan A, and Jordan F (2009). Reaction mechanisms of thiamin diphosphate enzymes: defining States of ionization and tautomerization of the cofactor at individual steps. FEBS J 276 (9), 2432–3446. doi:10.1111/j.1742-4658.2009.06964.x 19476485 PMC2795322

[R36] O’Brien TA, Kluger R, Pike DC, and Gennis RB (1980). Phosphonate analogues of pyruvate. Probes of substrate binding to pyruvate oxidase and other thiamin pyrophosphate-dependent decarboxylases. Biochimica Biophysica Acta (BBA) - Enzym 613 (1), 10–17. doi:10.1016/0005-2744(80)90186-2 6990987

[R37] Passalacqua KD, Charbonneau M-E, and O’Riordan MXD (2016). Bacterial metabolism shapes the host-pathogen interface. Microbiol. Spectr 4 (3). doi:10.1128/microbiolspec.VMBF-0027-2015 PMC4922512 27337445

[R38] Patel H, Nemeria NS, Brammer LA, Freel Meyers CL, and Jordan F (2012). Observation of thiamin-bound intermediates and microscopic rate constants for their interconversion on 1-deoxy-D-xylulose 5-phosphate synthase: 600-fold rate acceleration of pyruvate decarboxylation by D-glyceraldehyde-3-phosphate. J. Am. Chem. Soc 134 (44), 18374–18379. doi:10.1021/ja307315u 23072514 PMC3494461

[R39] Querol-Audí J, Boronat A, Centelles JJ, and Imperial S (2014). Catalytically important residues in E. Coli 1-deoxy-D-xylulose 5-phosphate synthase. J. Biosci. Med 02 (04), 30–35. doi:10.4236/jbm.2014.24006

[R40] Rodrıguez-Concepcion M, and Boronat A (2002). Elucidation of the methylerythritol phosphate pathway for isoprenoid biosynthesis in bacteria and plastids. A metabolic milestone achieved through genomics. Plant Physiol 130 (3), 1079–1089. doi:10.1104/pp.007138 12427975 PMC1540259

[R41] Rohmer L, Hocquet D, and Miller SI (2011). Are pathogenic bacteria just looking for food? Metabolism and microbial pathogenesis. Trends Microbiol 19 (7), 341–348. doi:10.1016/j.tim.2011.04.003 21600774 PMC3130110

[R42] Rohmer M, Knani M, Simonin P, Sutter B, and Sahm H (1993). Isoprenoid biosynthesis in bacteria: a novel pathway for the early steps leading to isopentenyl diphosphate. Biochem. J 295 (2), 517–524. doi:10.1042/bj2950517 8240251 PMC1134910

[R43] Sanders S, Bartee D, Harrison MJ, Phillips PD, Koppisch AT, and Freel Meyers CL (2018). Growth medium-dependent antimicrobial activity of early stage MEP pathway inhibitors. PloS One 13 (5), e0197638. doi:10.1371/journal.pone.0197638 29771999 PMC5957436

[R44] Sanders S, Vierling RJ, Bartee D, DeColli AA, Harrison MJ, Aklinski JL, (2017). Challenges and hallmarks of establishing alkylacetylphosphonates as probes of bacterial 1-Deoxy-d-Xylulose 5-phosphate synthase. ACS Infect. Dis 3 (7), 467–478. doi:10.1021/acsinfecdis.6b00168 28636325 PMC5650741

[R45] Schellenberger A (1998). Sixty years of thiamin diphosphate biochemistry. Biochimica Biophysica Acta (BBA) - Protein Struct. Mol. Enzym 1385 (2), 177–186. doi:10.1016/s0167-4838(98)00067-3 9655906

[R46] Schneider G, and Lindqvist Y (1998). Crystallography and mutagenesis of transketolase: mechanistic implications for enzymatic thiamin catalysis. Biochimica Biophysica Acta (BBA) - Protein Struct. Mol. Enzym 1385 (2), 387–398. doi:10.1016/s0167-4838(98)00082-x 9655943

[R47] Smith JM, Vierling RJ, and Meyers CF (2012). Selective inhibition of E. Coli 1-deoxy-D-xylulose-5-phosphate synthase by acetylphosphonates. MedChemComm 3, 65–67. doi:10.1039/c1md00233c 23326631 PMC3544079

[R48] Smith JM, Warrington NV, Vierling RJ, Kuhn ML, Anderson WF, Koppisch AT, (2014). Targeting DXP synthase in human pathogens: enzyme inhibition and antimicrobial activity of butylacetylphosphonate. J. Antibiotics 67 (1), 77–83. doi:10.1038/ja.2013.105 PMC3946878 24169798

[R49] Toci EM, Austin SL, Majumdar A, Woodcock HL, and Freel Meyers CL (2024). Disruption of an active site network leads to activation of C2α-lactylthiamin diphosphate on the antibacterial target 1-deoxy-D-xylulose-5-phosphate synthase. Biochemistry 63, 671–687. doi:10.1021/acs.biochem.3c00735 38393327 PMC11015862

[R50] Tong M, and Brown ED (2023). Food for thought: opportunities to target carbon metabolism in antibacterial drug discovery. Ann. N. Y. Acad. Sci 1524, 51–64. doi:10.1111/nyas.14991 37005709

[R51] Turner KH, Wessel AK, Palmer GC, Murray JL, and Whiteley M (2015). Essential genome of *Pseudomonas aeruginosa* in cystic fibrosis sputum. Proc. Natl. Acad. Sci 112 (13), 4110–4115. doi:10.1073/pnas.1419677112 25775563 PMC4386324

[R52] White JK, Handa S, Vankayala SL, Merkler DJ, and Woodcock HL (2016). Thiamin diphosphate activation in 1-Deoxy-d-Xylulose 5-phosphate synthase: insights into the mechanism and underlying intermolecular interactions. J. Phys. Chem. B 120 (37), 9922–9934. doi:10.1021/acs.jpcb.6b07248 27537621 PMC5379999

[R53] Wikner C, Meshalkina L, Nilsson U, Nikkola M, Lindqvist Y, Sundström M, (1994). Analysis of an invariant cofactor-protein interaction in thiamin diphosphate-dependent enzymes by site-directed mutagenesis. Glutamic acid 418 in transketolase is essential for catalysis. J. Biol. Chem 269 (51), 32144–32150. doi:10.1016/s0021-9258(18)31612-0 7798210

[R54] World Health Organization (2022). 2021 antibacterial agents in clinical and preclinical development: an overview and analysis Available at: https://apps.who.int/iris/bitstream/handle/10665/354545/9789240047655-eng.pdf?sequence=1.

[R55] Xiang S, Usunow G, Lange G, Busch M, and Tong L (2007). Crystal structure of 1-deoxy-D-xylulose 5-phosphate synthase, a crucial enzyme for isoprenoids biosynthesis. J. Biol. Chem 282 (4), 2676–2682. doi:10.1074/jbc.m610235200 17135236

[R56] Zhou J, Yang L, DeColli A, Freel Meyers C, Nemeria NS, and Jordan F (2017). Conformational dynamics of 1-Deoxy-d-Xylulose 5-phosphate synthase on ligand binding revealed by H/D exchange MS. Proc. Natl. Acad. Sci 114 (35), 9355–9360. doi:10.1073/pnas.1619981114 28808005 PMC5584406

