# Supplementary material for: An activity-based probe for antimicrobial target DXP synthase, a thiamin diphosphate-dependent enzyme
Source: Front Chem Biol. Author manuscript; Available in PMC 2024 Nov 14. (PMC11562961; doi:10.3389/fchbi.2024.1389620)
Supplement: Supplementary Material [file NIHMS2022191-supplement-Supplementary_Material.docx]

Supplementary Material

**An activity-based probe for antimicrobial target DXP synthase, a thiamine diphosphate-dependent enzyme**

Lauren B Coco^1^ and Caren L Freel Meyers^1*^

^1^Johns Hopkins University School of Medicine, Department of Pharmacology and Molecular Sciences, Baltimore, Maryland, United States.

*** Correspondence:**Caren Freel Meyers
[cmeyers@jhmi.edu](mailto:cmeyers@jhmi.edu)

**Table of Contents**

**1 Supplementary Figures and Tables**

**Figure S1.** Ultraviolet absorbance profile of ABP **1** 2

**Figure S2.** Control experiments confirming **1** does not crosslink DXPS during the coupled assay and does not inhibit the coupling system. 3

**Figure S3.** Circular dichroism experiments showing PLThDP formation from **1** 4

**Figure S4.** Labeling of DXPS by **1** and controls omitting key reaction components 5

**Figure S5.** Dose-response DXPS labeling experiments 6

**Figure S6.** Secondary structure of *Ec*E370A DXPS using circular dichroism 7

**Figure S7.** Stability of *Ec*E370A DXPS compared to wild-type DXPS using circular dichroism 8

**Figure S8.** *Ec*E370A DXPS and denatured wild-type DXPS labeling by **1** 9

**Figure S9.** Competition assays of **1** with DXPS inhibitors **7**, **8**, and **9** 10

**Figure S10.** Assessment of off-target effects of **1** on PDH and PDC 11

**Table S1.** Initial velocities and % enzyme activity of PDH and PDC in the presence **1** 12

**Figure S11.** Evaluation of off-target and non-specific labeling by **1** 13

**Figure S12.** ABP **1** labeling of DXPS in bacterial lysate and competition with **9** 14

**2 Chemistry**

**2.1** Synthesis of 2-(3-(but-3-yn-1-yl)-3H-diazirin-3-yl)ethan-1-ol (**4**) 15

**2.2** Synthesis of 2-(3-(but-3-yn-1-yl)-3H-diazirin-3-yl)ethyl dimethyl phosphite (**5**) 15

**2.3** Synthesis of 2-(3-(but-3-yn-1-yl)-3H-diazirin-3-yl)ethyl acetylphosphonate (**1**) 15

**3 Compound characterization**

**3.1** NMR (^1^H and ^31^P) spectra for compounds **1**, **4**, **5**  16

**3.2** High resolution mass spectrometry (HRMS) of **1** 19

**3.3** HPLC chromatogram of **1** 19

**1 Supplementary Figures and Tables**

**Supplementary Figure 1.** Ultraviolet (UV) absorbance profile of ABP **1** showing characteristic diazirine absorbance at 340 nm (red line) compared to a water blank (blue line).

**
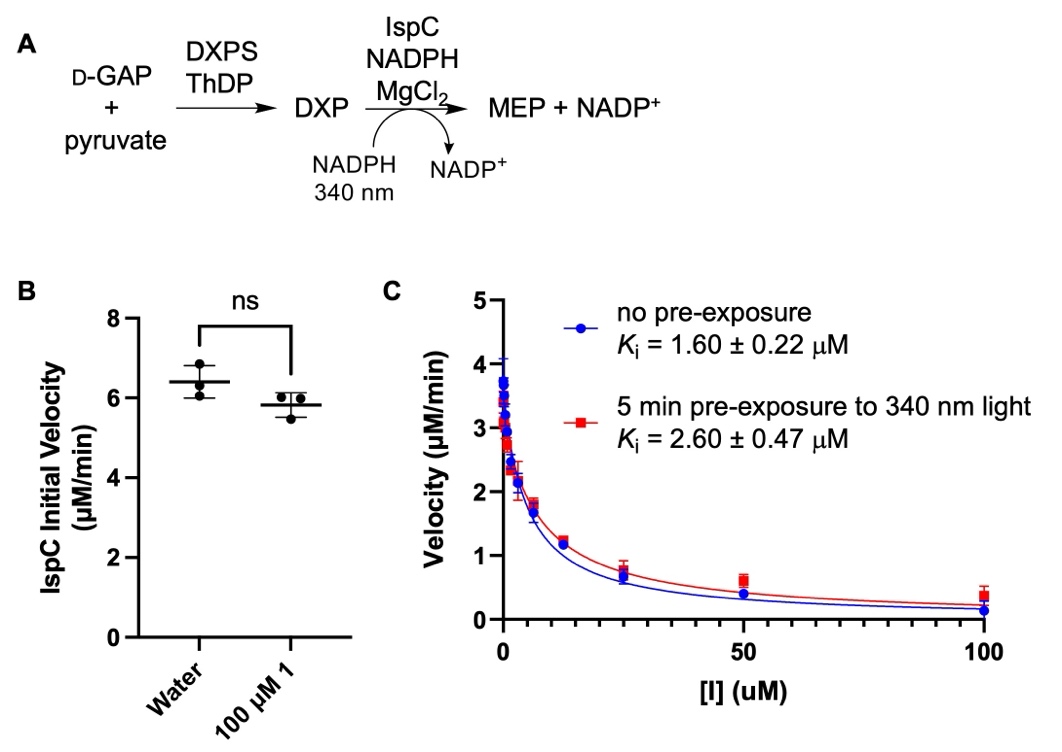
**

**Supplementary Figure 2.** Control experiments confirming **1** does not crosslink DXPS during the coupled assay and does not inhibit the coupling system. **A**) Scheme of the DXPS-IspC coupled assay. Initial velocity of DXP formation is determined by measuring rate of NADPH depletion at 340 nm. **B**) Plot of IspC initial velocity in the presence or absence of **1**. No significant difference was observed in *E. coli* IspC initial velocity in the presence of 100 μM **1**, the highest [**1**] used to determine *K*_i_. Statistical significance was determined using an unpaired t test. *Experimental procedure*: DXP was prepared by incubating DXPS (1 μM) in buffer (2 mM MgCl_2_, 5 mM NaCl, 1 mM ThDP, 100 mM HEPES pH 8) for 1 hour at 37 °C following the addition of substrates (4 mM pyruvate and D-GAP). DXPS was then removed using a centrifugal filter (10 kDa molecular weight cutoff, 10 minutes, 13000 ×g). The eluate containing DXP (0, 2, 4, 6, and 8 μL) was added to 320 μL of IspC (2 μM) in buffer (2 mM MgCl_2_, 100 mM HEPES pH 8, 200 μM NADPH). The consumption of NADPH was measured at 340 nm after 30 minutes at 25 °C and used to quantify [DXP]. To determine if **1** inhibits IspC, IspC (100 nM) was pre-incubated in buffer (2 mM MgCl_2_, 100 mM HEPES pH 8, 200 μM NADPH) for 10 minutes at 25 °C with **1** (100 μM) or water. The reaction was initiated by the addition of 196 μM DXP (2×*K*_m_). The initial velocity of DXP conversion in the presence of **1** or water was determined by measuring the rate of NADPH depletion at 340 nm. Experiments were performed in triplicate. **C**) Plot of DXPS velocity as a function of [**1**] with and without pre-exposure of the enzyme-**1** mixture to 340 nm light (5 minutes, 25 °C) in a plate reader. *K*_i_ values were determined under each condition using the Morrison equation (see Materials and Methods). The data indicate there is no significant difference in potency of **1** with or without pre-exposure to 340 nm light. Kinetic experiments were performed in triplicate at 25 °C. Error bars represent standard deviation. ns = no significance.


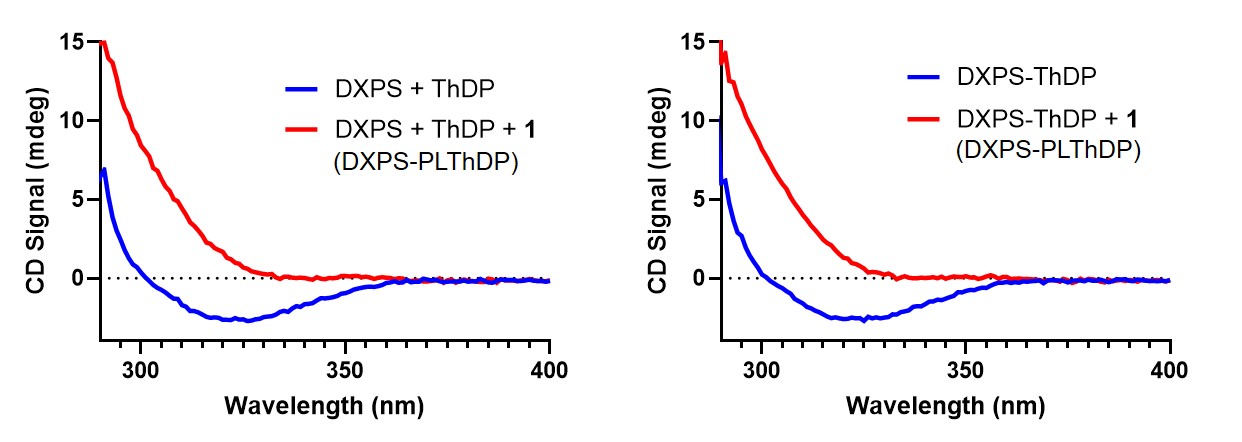


**Supplementary Figure 3.** Replicate circular dichroism (CD) experiments showing PLThDP formation from **1** (50 μM) on *Ec*DXPS (30 μM). CD traces are the average of two scans. Experiments performed at 25 °C. (*n* = 2)


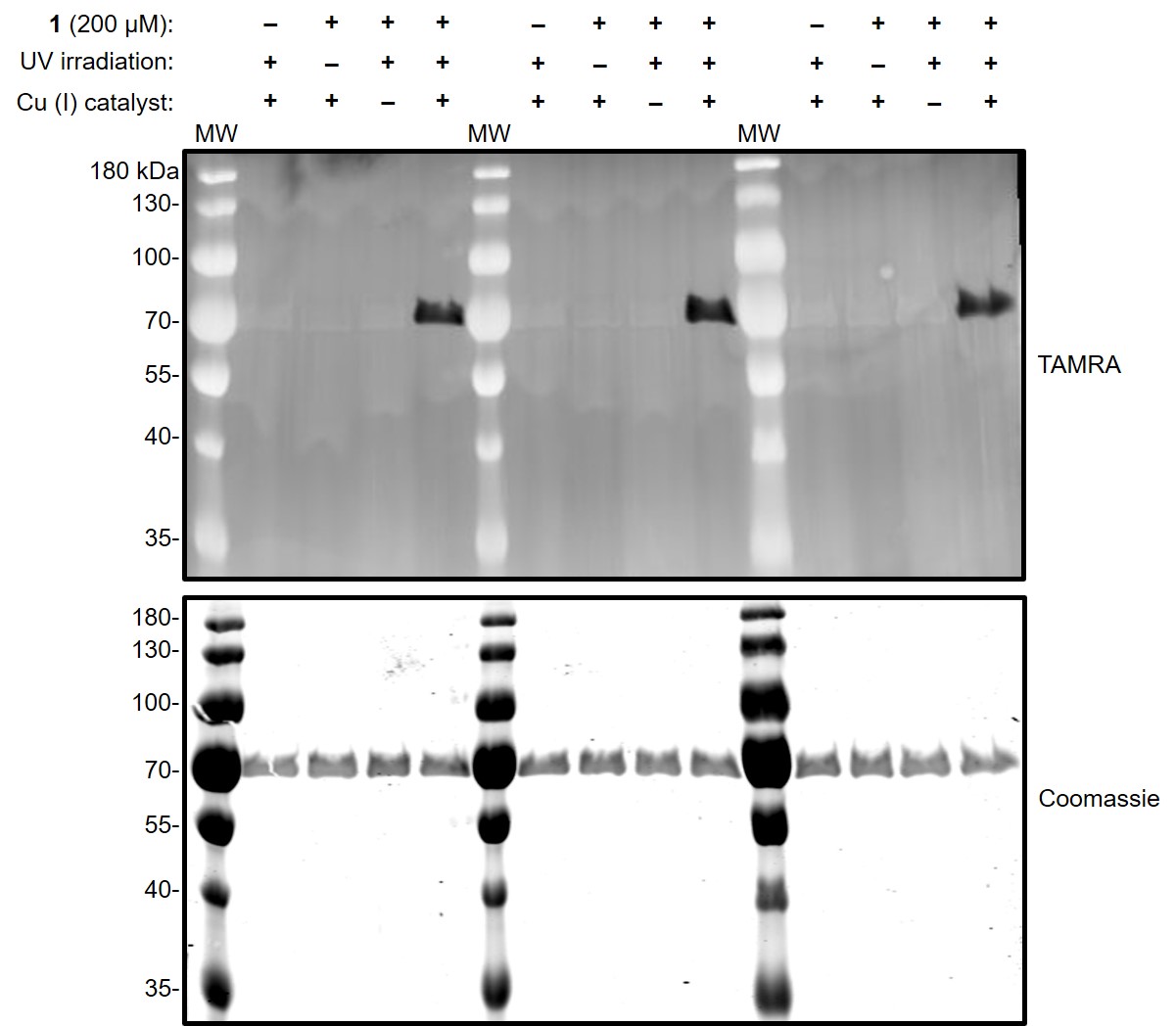


**Supplementary Figure 4.** Experimental replicates for labeling of DXPS (3 μM) by **1** (200 μM) and controls omitting key reaction components (**1**, UV irradiation at 365 nm and Cu(I)). Gel images were prepared using ImageJ. TAMRA = fluorescent gel scan; MW = protein molecular weight marker (*n* = 3)

**
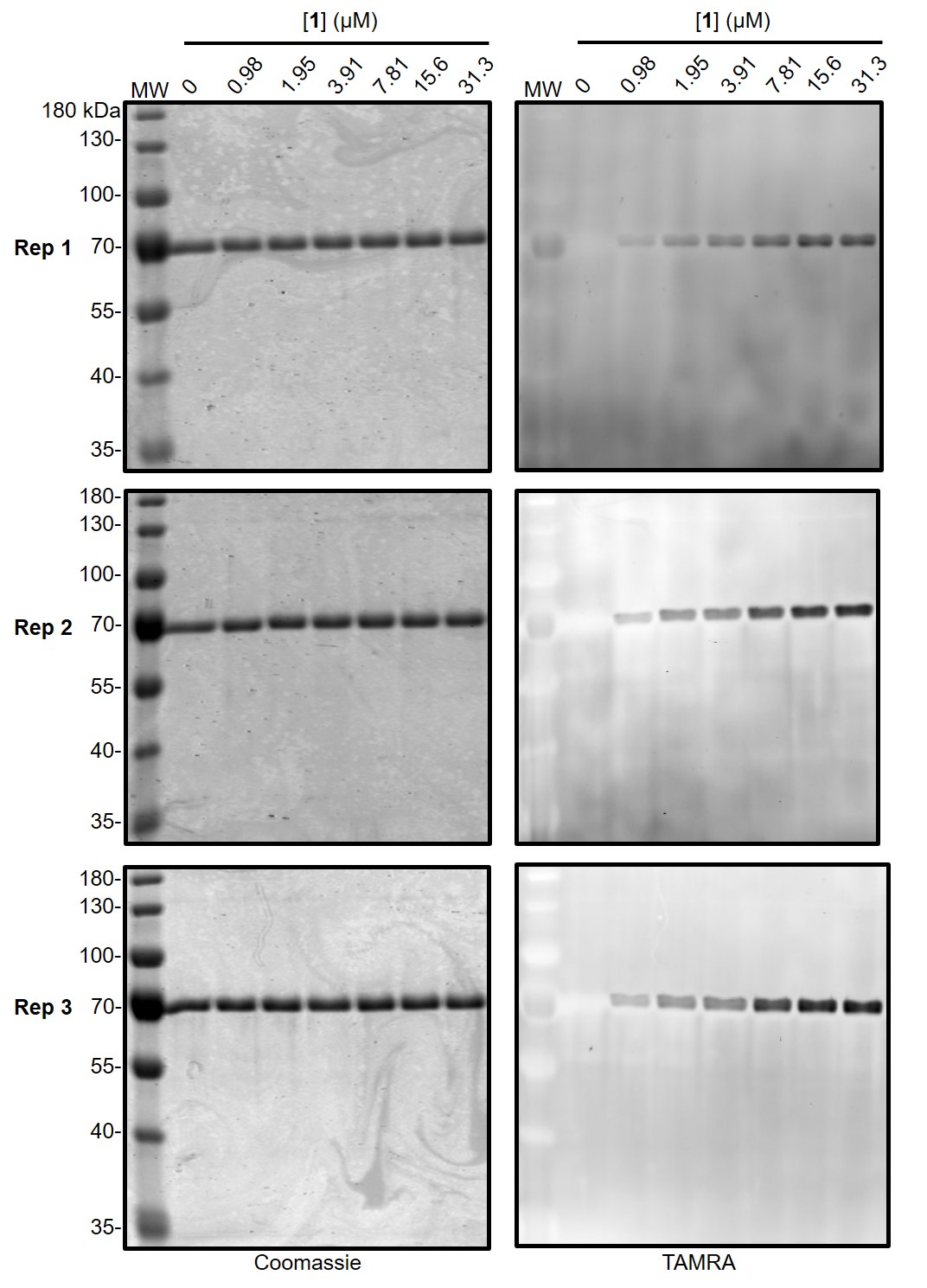
**

**Supplementary Figure 5.** Experimental replicates of dose-response DXPS labeling experiments. DXPS (3 μM) was incubated with **1** at varying concentrations, and then irradiated (365 nm, 180 W) for 3 minutes at 4 °C. Gel images were prepared and quantified using ImageJ. MW = protein molecular weight marker; TAMRA = fluorescent gel scan. (*n* = 3)


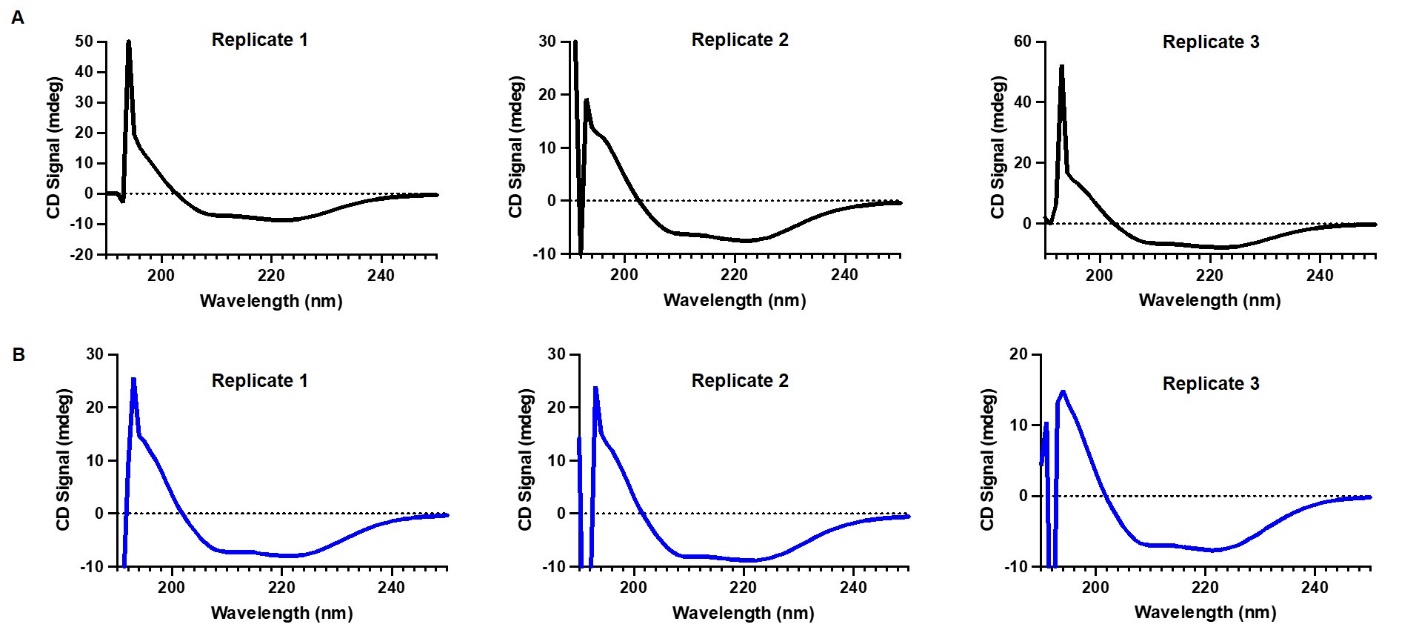


**Supplementary Figure 6.** Secondary structure of *Ec*E370A DXPS (blue, **B**) compared to wild-type DXPS (black, **A**) using circular dichroism (CD). DXPS was first exchanged into a CD-compatible buffer (25 mM Tris pH 8, 10 μM ThDP, 1 mM MgCl_2_, 100 mM NaCl); concentrated DXPS was diluted 10-fold in CD buffer and concentrated using a 30 kDa molecular weight cutoff centrifugal filter. Following buffer exchange, protein concentration was determined by denaturing 100 μL protein mix in 400 μL 7.5 M guanidine-HCl and measuring A_280_. DXPS was then diluted to 1 μM in CD buffer in a final volume of 130 μL. CD scans were performed from 180-250 nm, 1 nm step, 2 second averaging time. Experiments were performed at 25 °C using a 0.5 mm quartz cuvette. (*n* = 3)


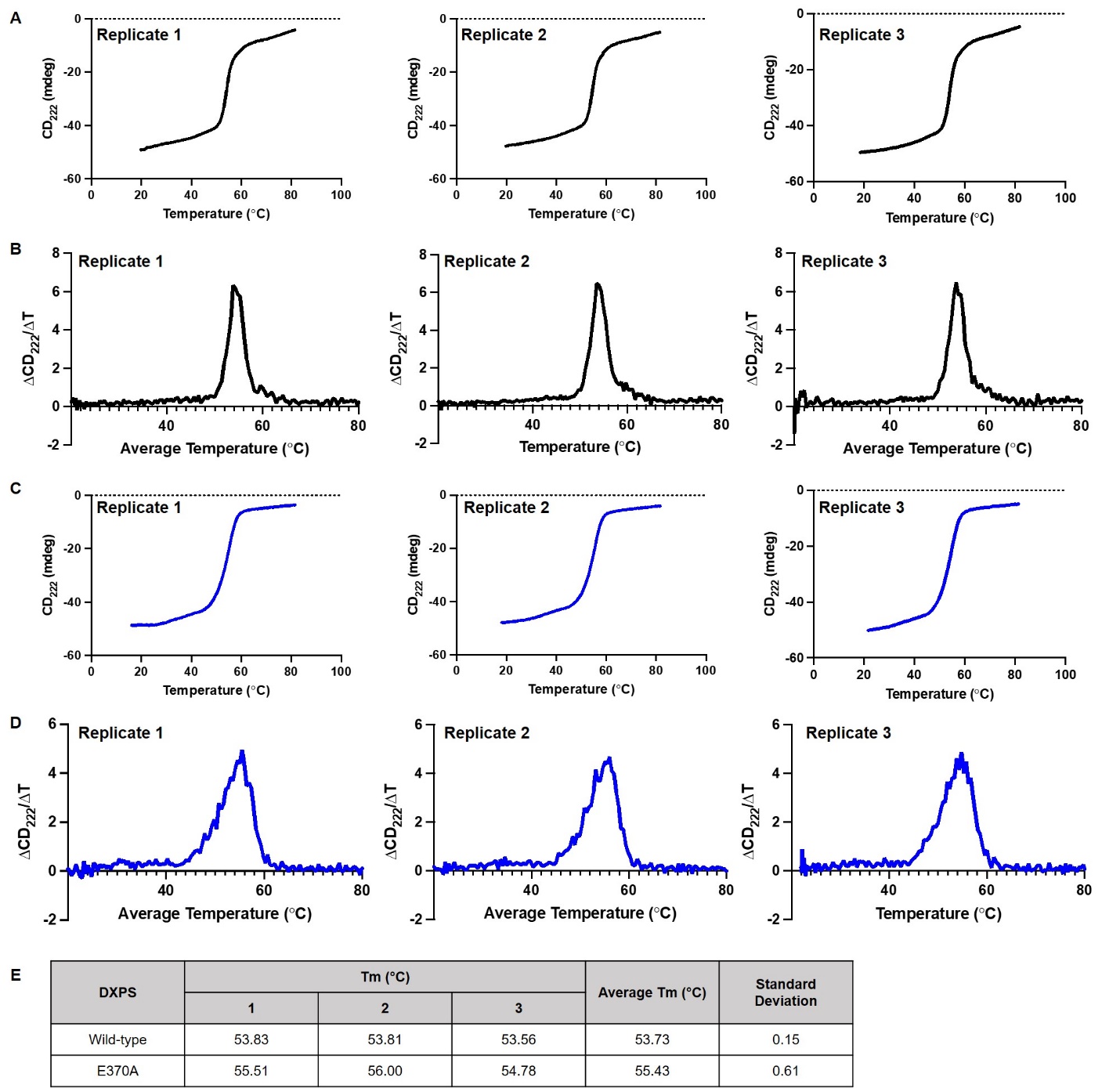


**Supplementary Figure 7.** Stability of *Ec*E370A DXPS (blue, **C** & **D**) was compared to wild-type DXPS (black, **A** & **B**) using circular dichroism (CD). Replicate melting curves for wild-type (**A**) and E370A (**C**) and corresponding first derivatives for wild-type (**B**) and E370A (**D**) are shown. The protein melting temperature (Tm) for E370A DXPS is comparable to wild-type DXPS. (**E**) Tm summary. Tm values were calculated from the curve maximum of the first derivatives. DXPS was first exchanged into a CD-compatible buffer (25 mM Tris pH 8, 10 μM ThDP, 1 mM MgCl_2_, 100 mM NaCl); concentrated DXPS was diluted 10-fold in CD buffer and concentrated using a 30 kDa molecular weight cutoff centrifugal filter. Following buffer exchange, protein concentration was determined by denaturing 100 μL protein mix in 400 μL 7.5 M guanidine-HCl and measuring A_280_. DXPS was then diluted to 3 μM in CD buffer (final volume 2.5 mL). CD scans were performed as such: temperature range 20-88 °C, 0.5 °C step, 1 °C/min, tolerance 0.2 °C, 20 sec setting time, 20 sec per point, measured at 222 nm. Experiments were performed using a 1 cm quartz cuvette. (*n* = 3)

**
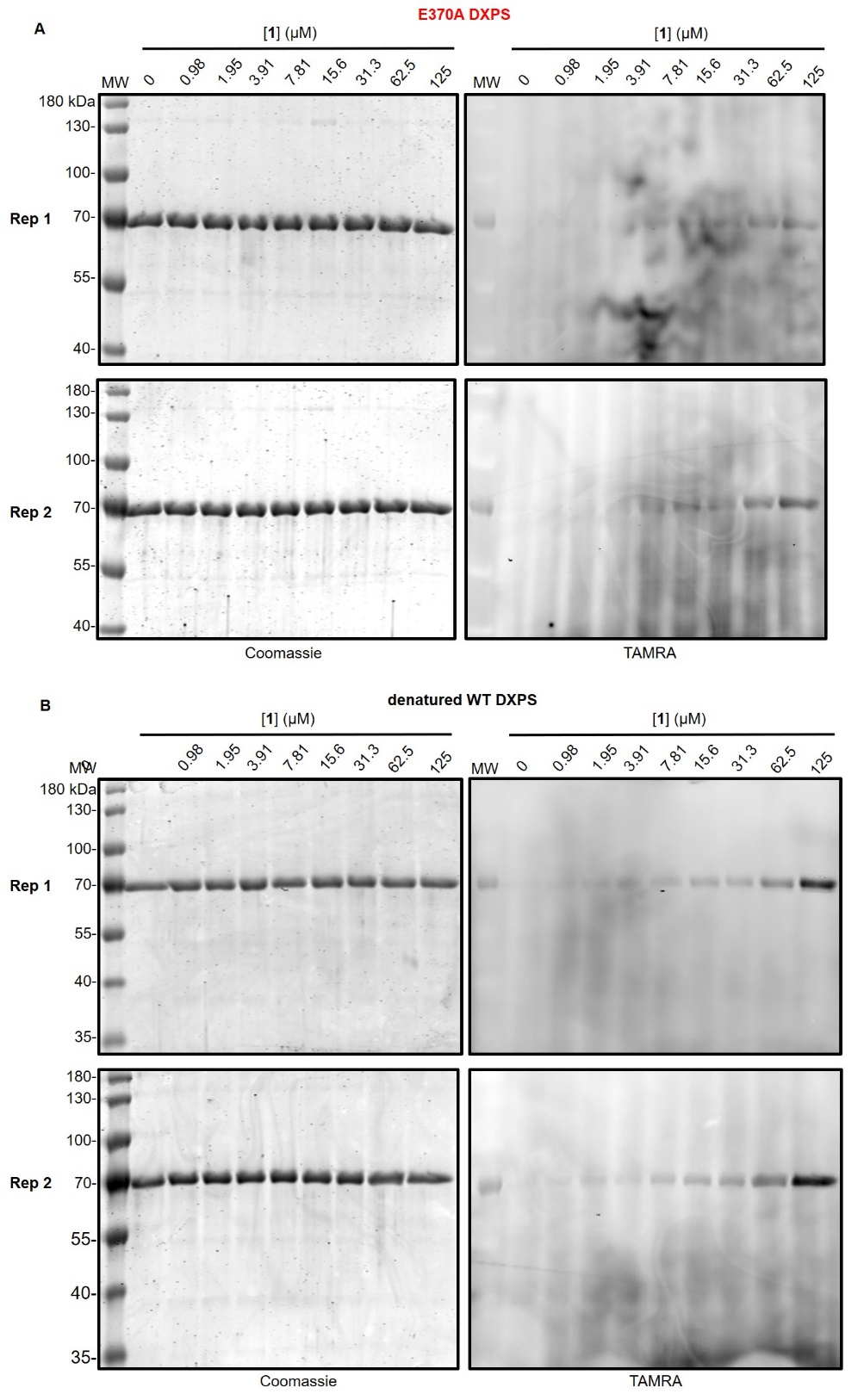
**

**Supplementary Figure 8.** Experimental replicates of *Ec*E370A DXPS and denatured wild-type (WT) DXPS labeling by **1**. **A**) Labeling of E370A DXPS. **B**) Labeling of denatured WT DXPS. Minimal labeling was observed with inactive DXPS samples in each case up to 7.81 mM **1**, consistent with a requirement for active DXPS for efficient labeling. At [**1**] > 31.3 mM, non-specific labeling is more pronounced. In all cases, 3 μM enzyme was used, and mixtures of enzyme and **1** were irradiated (365 nm, 180 W) for 3 minutes at 4 °C. Gel images were prepared and quantified using ImageJ. MW = protein molecular weight marker. TAMRA = fluorescent gel scan (*n* = 2)


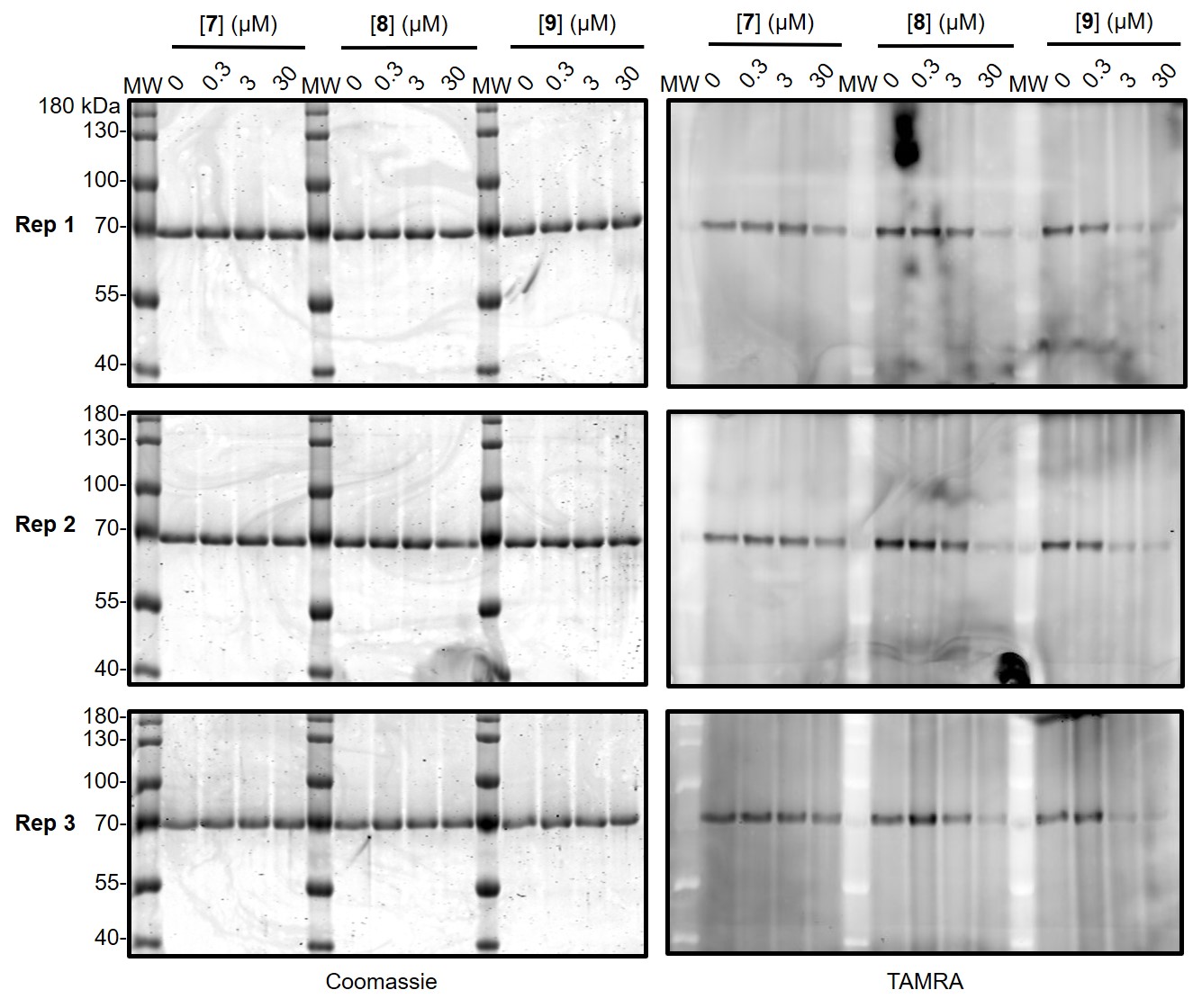


**Supplementary Figure 9.** Experimental replicates from competition assays of **1** with DXPS inhibitors **7**, **8**, and **9**. All samples held [DXPS] (3 μM) and [**1**] (50 μM) constant. Gel images were prepared and quantified using ImageJ. MW = protein molecular weight marker. TAMRA = fluorescent gel scan (*n* = 3)


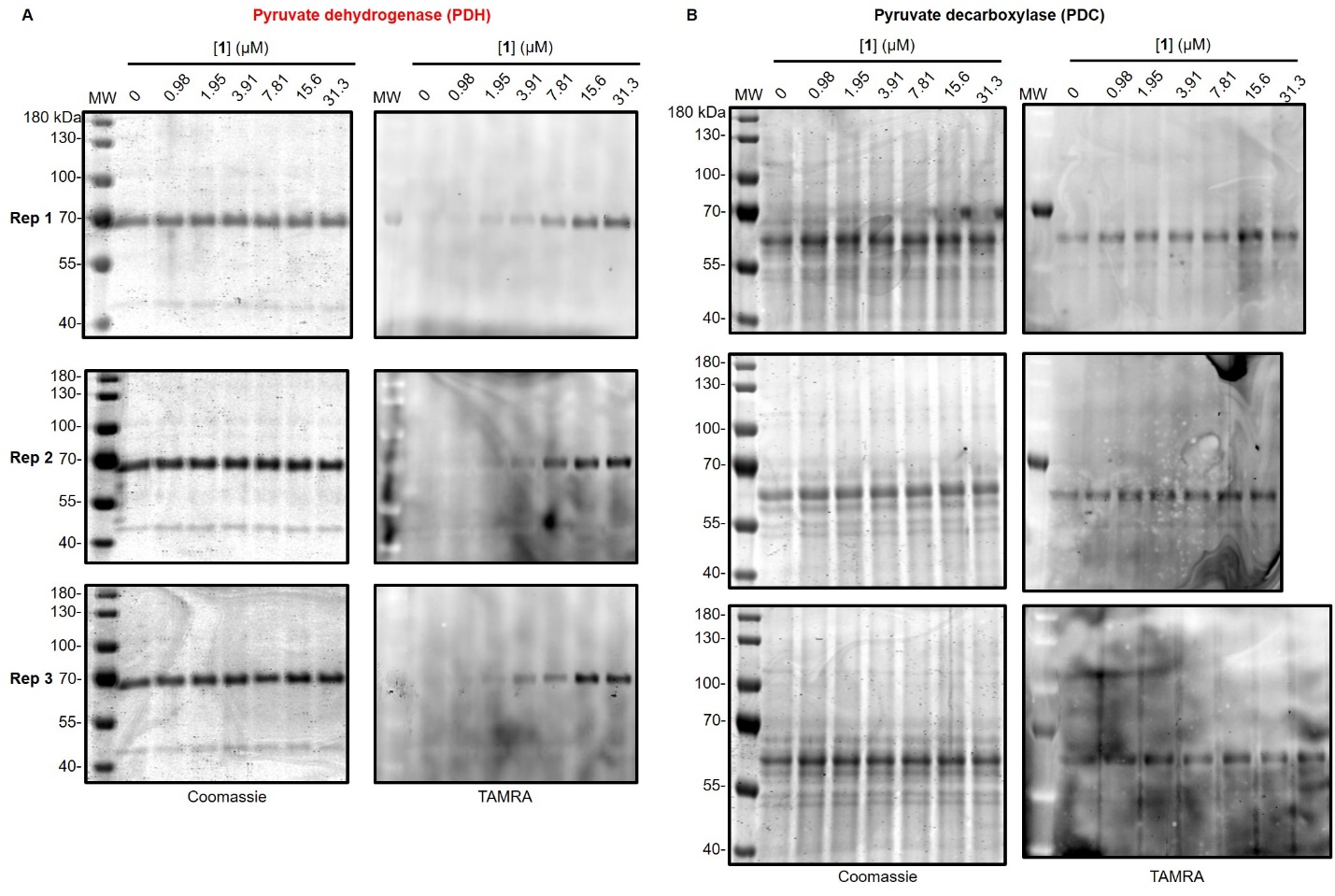


**Supplementary Figure 10.** Experimental replicates for assessment of off-target effects of **1** on ThDP-dependent enzymes PDH and PDC. **A**) Labeling of PDH by **1**. **B**) Labeling of PDC by **1**. Experiments were performed with 3 μM enzyme. In each case, enzyme was incubated with **1** for 10 minutes on ice and then samples were irradiated for 3 minutes at 4 °C. ImageJ was used for generating gel images and quantifying pixel density. MW = protein molecular weight marker. TAMRA = fluorescent gel scan. (*n* = 3)


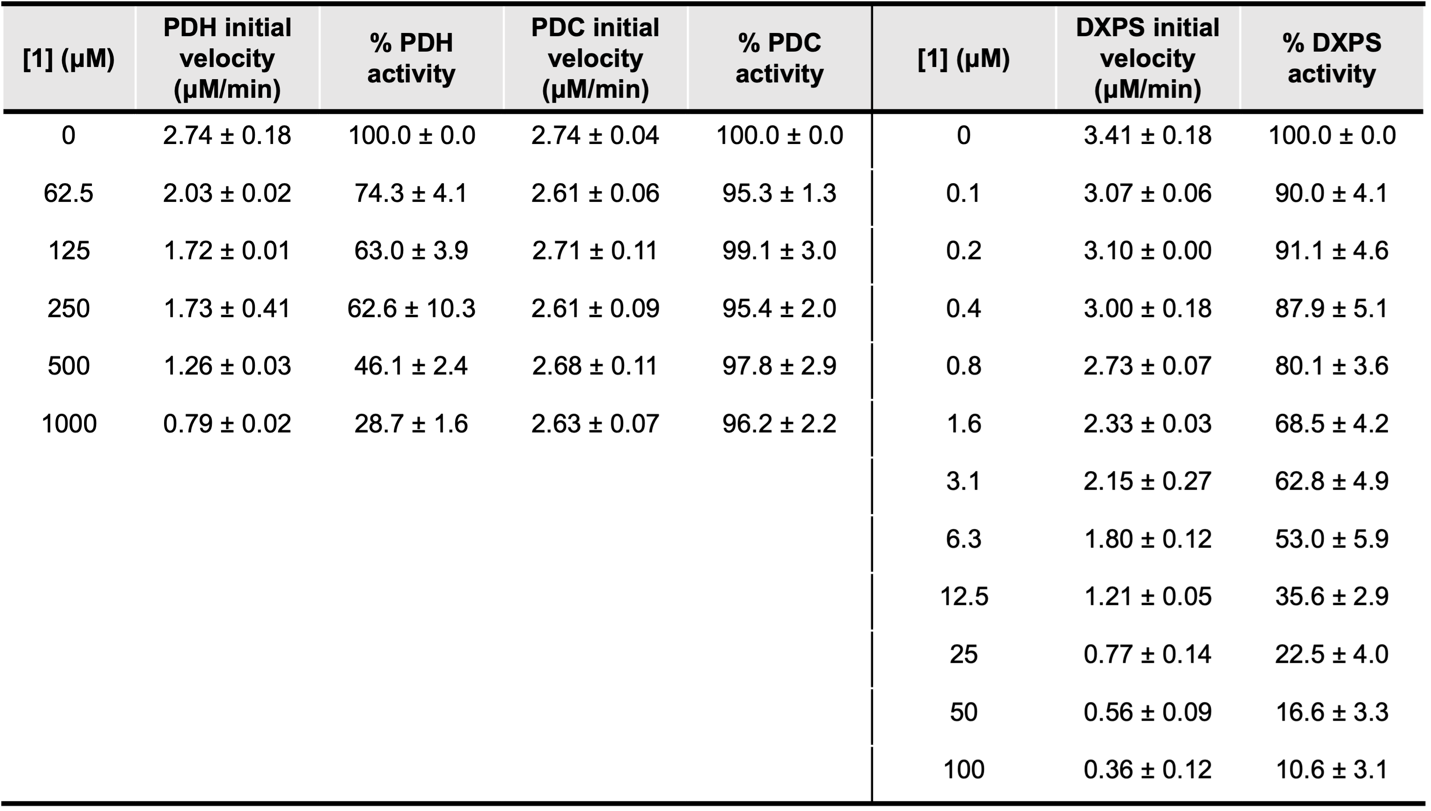


**Supplementary Table 1.** Summary of initial velocities and % enzyme activity of ThDP-dependent PDH (porcine heart), PDC (*Saccharomyces cerevisiae*) and *E. coli* DXPS in the presence of varying concentrations of ABP **1** up to 1000 μM (for PDH and PDC), or up to 100 μM (DXPS). These data for DXPS activity were used to generate the Morrison *K*_i_ curve in **Figure 4C** and **Supplementary Figure 2C**. Standard deviation was determined from kinetic experiments performed in triplicate. See Materials and Methods for reaction conditions.


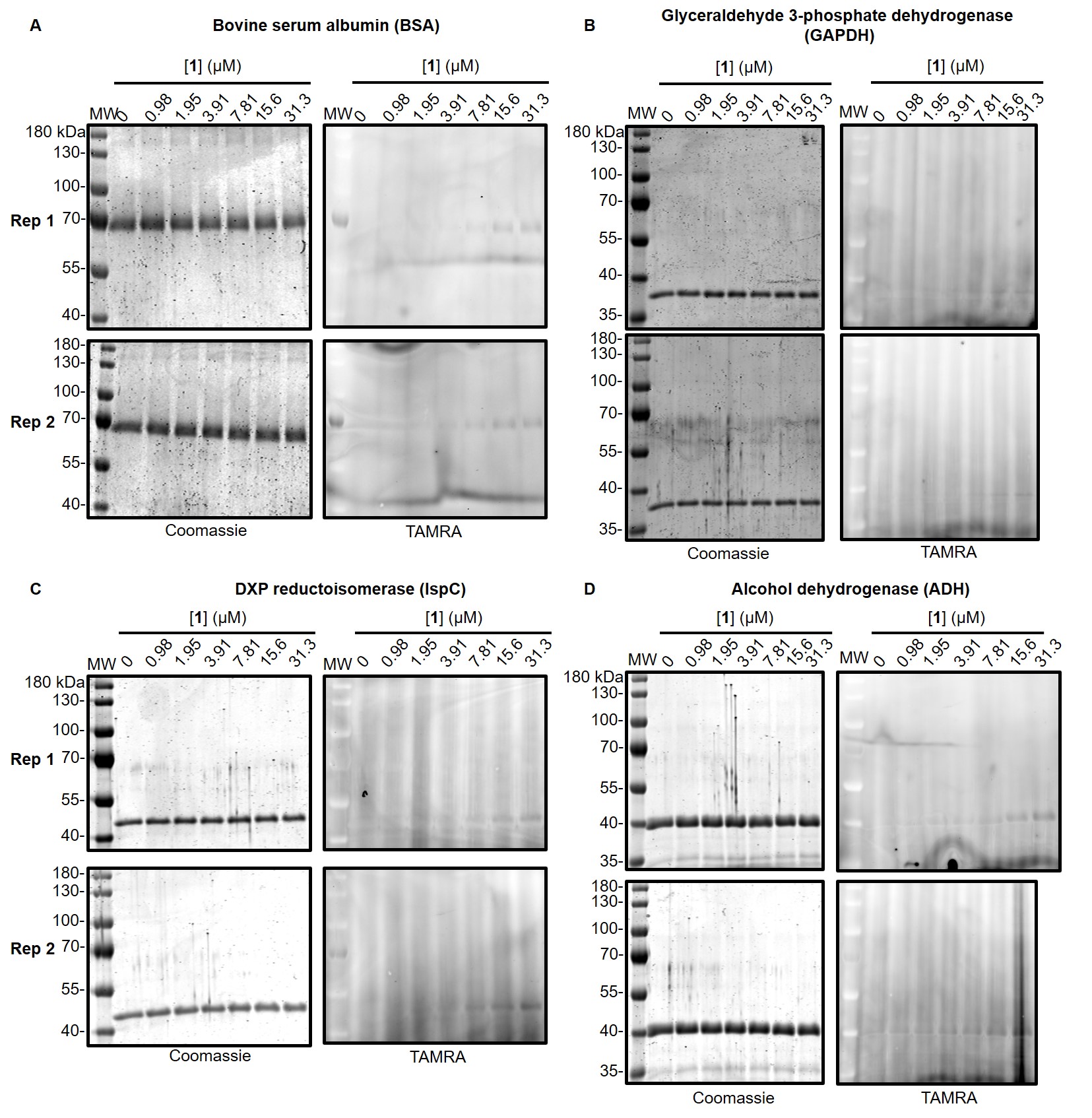


**Supplementary Figure 11.** Experimental replicates for evaluation of off-target and non-specific labeling by **1**. Replicates are shown for non-specific labeling of **A**) bovine serum albumin (BSA), **B**) glyceraldehyde 3-phosphate dehydrogenase (GAPDH), **C**) DXP reductoisomerase (IspC), and(**D**) alcohol dehydrogenase (ADH). Protein was maintained at 3 μM in all cases. Mixtures of protein and **1** were irradiated (365 nm) for 3 minutes at 4 °C. Gel images were prepared and quantified using ImageJ. MW = protein molecular weight marker. TAMRA = fluorescent gel scan. (*n* = 2) *Abbreviations*: Bovine serum album (BSA), glyceraldehyde 3-phosphate (GAPDH, rabbit muscle), IspC (*E. coli*), alcohol dehydrogenase (ADH, *Saccharomyces cerevisiae*), pyruvate dehydrogenase (PDH, porcine heart).


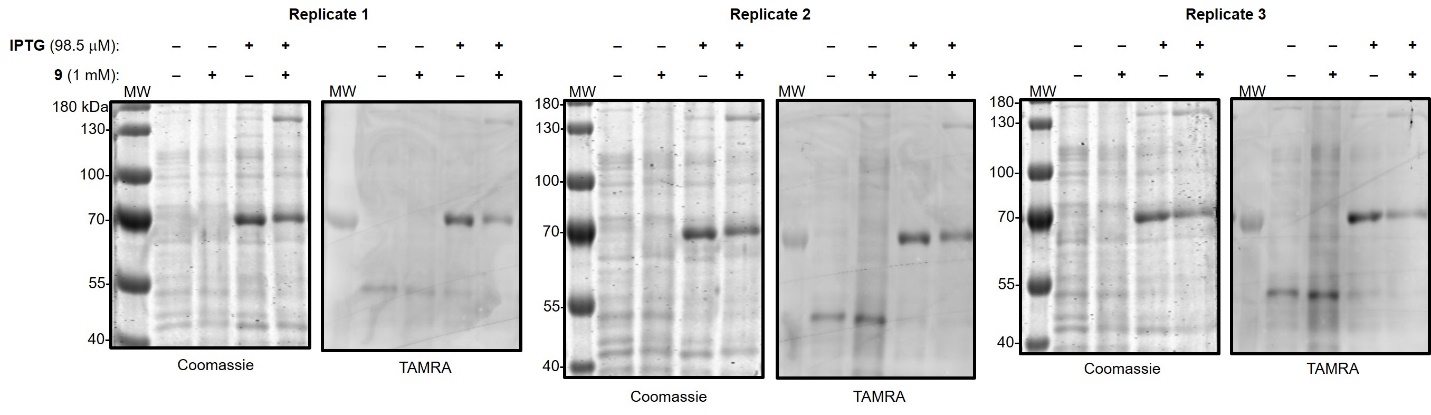


**Supplementary Figure 12.** Replicate experiments showing that **1** labels active DXPS in bacterial lysate, and **9** reduces labeling of DXPS by **1**. (*n* = 3)

**2 Chemistry**

**2.1 Synthesis of 2-(3-(but-3-yn-1-yl)-3*H*-diazirin-3-yl)ethan-1-ol (4).**

Anhydrous gaseous ammonia (~ 60 mL) was condensed into a pressure flask cooled to -78 °C. 1-Hydroxyhept-6-yn-3-one (**2**, 7.93 mmol, 1 eq, Ambeed) was added to the condensed ammonia at -78 °C. The reaction flask was capped at -78 °C and slowly brought to ambient temperature. (Note: pressure builds in the closed flask upon warming and thus the reaction flask should be in a chemical fume hood and behind a blast shield for safety.) The reaction proceeded for 5 hours at ambient temperature, and was then cooled to -78 °C and the flask was opened. Hydroxylamine *O*-sulfonic acid (HOSA, 8.72 mmol, 1.1 eq) in anhydrous methanol (1.41 M, 6.2 mL) was added dropwise at -78 °C. The flask was capped again and brought to ambient temperature. The reaction proceeded for 1 hour at ambient temperature, was cooled to -78 °C and the flask was opened. The reaction mixture was allowed to slowly warm to ambient temperature, open to atmosphere. (Note: at this stage, the blast shield is no longer needed.) The flask was left open to atmosphere in a chemical fume hood overnight to allow for excess ammonia to evaporate. Methanol (25 mL) was added to the slurry the following day, and insoluble ammonium salt reaction byproducts were removed by vacuum filtration. The methanol filtrate containing diaziridine **3** was collected and was used without further characterization or manipulation and assuming 100 % yield. The filtrate was cooled on ice. Anhydrous TEA (1.11 mL, 7.93 mmol, 1 eq) was added to diaziridine **3** (in methanol filtrate) followed by addition of iodine (1.208 g, 4.76 mmol, 0.6 eq) in small portions on ice. A brown color remained following addition of the final portion of I_2_. Methanol was removed under vacuum and an aqueous solution of saturated sodium thiosulfate (75 mL) followed by diethyl ether (75 mL) were added to the remaining solid. Crude product was extracted with diethyl ether (3 × 75 mL). Ether fractions were combined, dried over MgSO_4_, filtered and concentrated to give diazirine **4**, which was purified by flash chromatography (30 % ethyl acetate and hexanes) in the absence of UV detection. Fractions were assessed by thin layer chromatography, combined (30 % ethyl acetate and hexanes; KMnO_4_ stain; Rf 0.3) and concentrated under vacuum to give 659 mg **4** as a colorless oil in 60 % yield over two steps. ^1^H NMR (500 MHz, CHLOROFORM-*D*) δ 3.50 (q, *J* = 5.7 Hz, 2H), 2.05 (td, *J* = 7.3, 2.5 Hz, 2H), 2.00 (t, *J* = 2.6 Hz, 1H), 1.75 – 1.66 (m, 4H). NMR spectra are provided below.

**2.2 Synthesis of 2-(3-(but-3-yn-1-yl)-3H-diazirin-3-yl)ethyl dimethyl phosphite (5).**

A flask containing **4** (659 mg, 4.77 mmol, 1 eq) was flushed with argon. Tetrazole was added dropwise to **4** (10.6 mL, 0.45 M in acetonitrile, 1 eq) and then dimethyl-*N*,*N*-diisopropylphosphoramidite (1.10 mL , 4.77 mmol, 1 eq, Chem-Impex) was added very slowly dropwise at ambient temperature under argon. The reaction mixture stirred for 2 hours at ambient temperature. Dichloromethane (50 mL) was added to the reaction mixture, and insoluble salts were removed by vacuum filtration. The filtrate was washed 3 times with 1 M NaOH (50 mL). The organic layer was collected, dried over MgSO_4_, filtered, and concentrated under vacuum to give 1.03 g of **5** as a colorless oil in 94 % yield. ^1^H NMR (500 MHz, CHLOROFORM-*D*) δ 3.71 – 3.65 (m, 2H), 3.54 (dd, *J* = 10.8, 0.9 Hz, 5H), 2.05 – 2.00 (m, 3H), 1.98 (t, *J* = 2.6 Hz, 1H), 1.74 – 1.68 (m, 4H). ^31^P NMR (202 MHz, CHLOROFORM-*D*) δ 115.20. NMR spectra are provided below.

**2.3 Synthesis of 2-(3-(but-3-yn-1-yl)-3H-diazirin-3-yl)ethyl acetylphosphonate (1).**

A flame-dried flask was charged with acetyl chloride (1.6 mL, 22.6 mmol, 5 eq) under argon. Phosphite **5** (1.03 g, 4.47 mmol, 1 eq) was added very slowly, dropwise over 20 minutes at ambient temperature. Following addition of **5**, the reaction proceeded for 30 minutes at ambient temperature under argon. Excess acetyl chloride was removed under vacuum to give acetylphosphonate diester **6** as an oil, which was subsequently dissolved in anhydrous acetonitrile (1 M, 4.47 mL) under argon. Lithium bromide (dry and stored in an oven at 130 °C) was quickly weighed and added to the reaction mixture. The reaction proceeded for 16 hours with stirring at ambient temperature under argon. ABP **1** precipitated over this time, and was collected by vacuum filtration, washed several times with acetonitrile and dried under vacuum to give 588 mg of **1** a white solid in 53 % yield over two steps. ^1^H NMR (500 MHz, D_2_O) δ 3.83 – 3.76 (m, 2H), 2.47 – 2.41 (m, 3H), 2.41 – 2.34 (m, 1H), 2.10 – 2.04 (m, 2H), 1.82 – 1.70 (m, 5H). ^31^P NMR (202 MHz, D_2_O) δ -26.76. ABP **1** is 85 % pure by ^31^P NMR. HRMS (ESI-TOF) m/z calculated for C_9_H_12_N_2_O_4_P [M-H]^-^ 243.0540; found: 243.0543. Purity of **1** was determined by reversed-phase (RP) high performance liquid chromatography (HPLC) with detection at 340 nm (diazirine λ_max_). A fresh 1 mM solution of **1** in water was prepared immediately prior to injection onto the RP column. Then, 5 μL of the 1 mM inhibitor solution was injected onto a ZORBAX 80Å extend-C18 column (4.6 x 50 mm, 3.5 μm) and eluted at a flow rate of 1 mL/min. Mobile phases used include water + 0.1% trifluoroacetic acid (solvent A) and acetonitrile (solvent B). The following method was used: 30 seconds at 5% B, go to 95% B over 4 minutes, and hold 95% B for 54 seconds. ABP **1** is 95 % pure by HPLC. NMR spectra, high resolution mass spectrometry (HRMS), and HPLC analyses are provided below.

**3 Compound characterization**

**3.1 NMR (^1^H and ^31^P) spectra
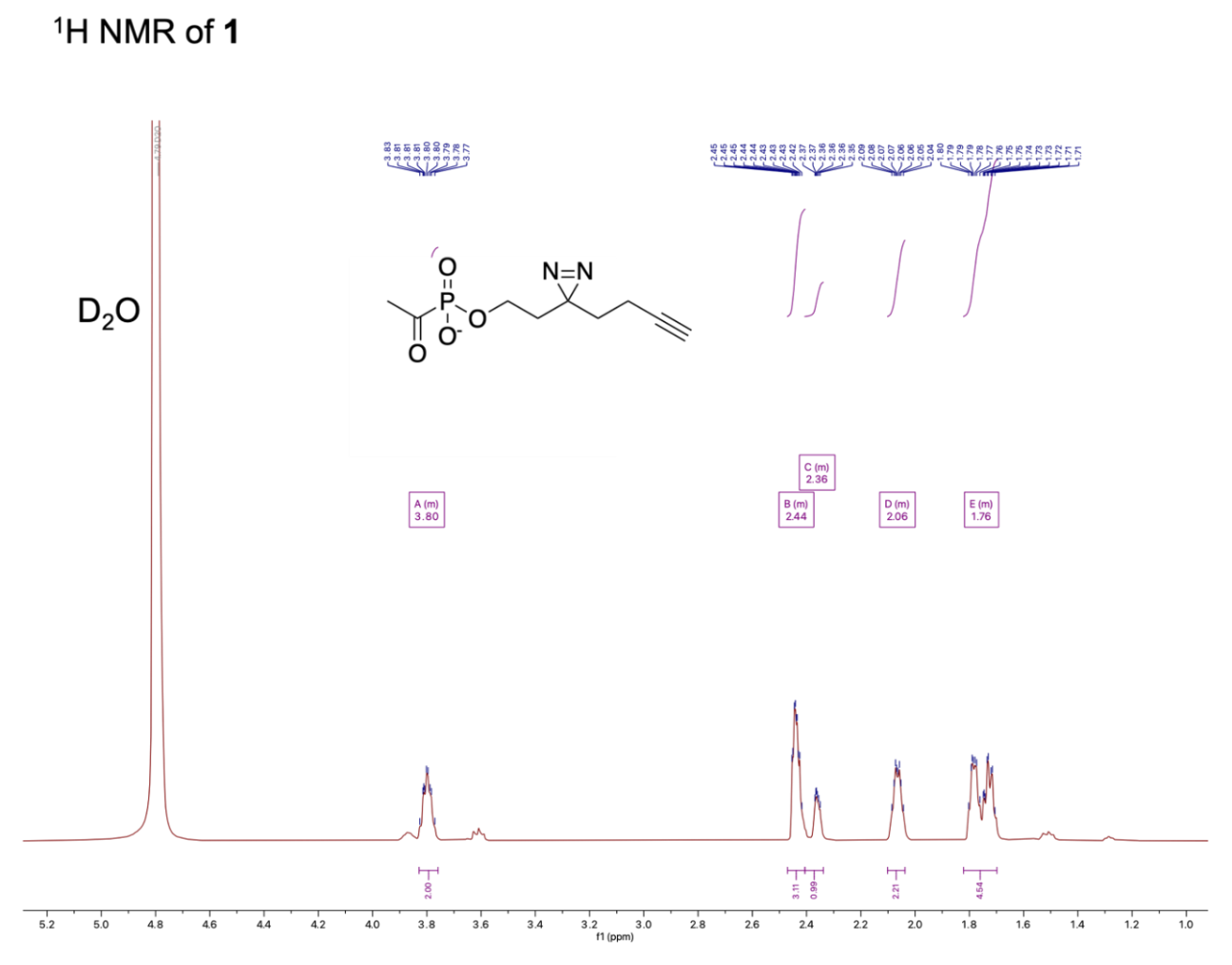

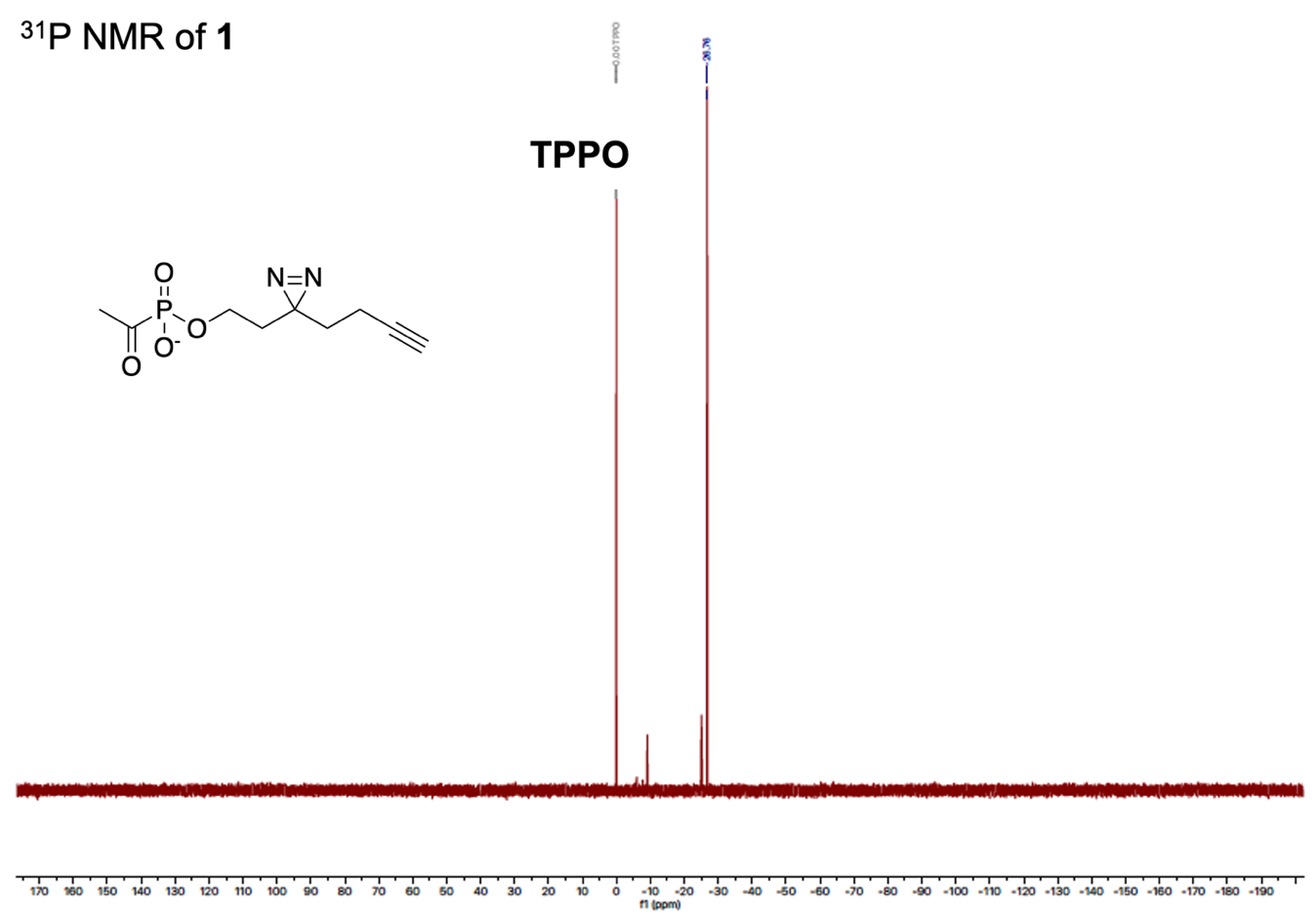

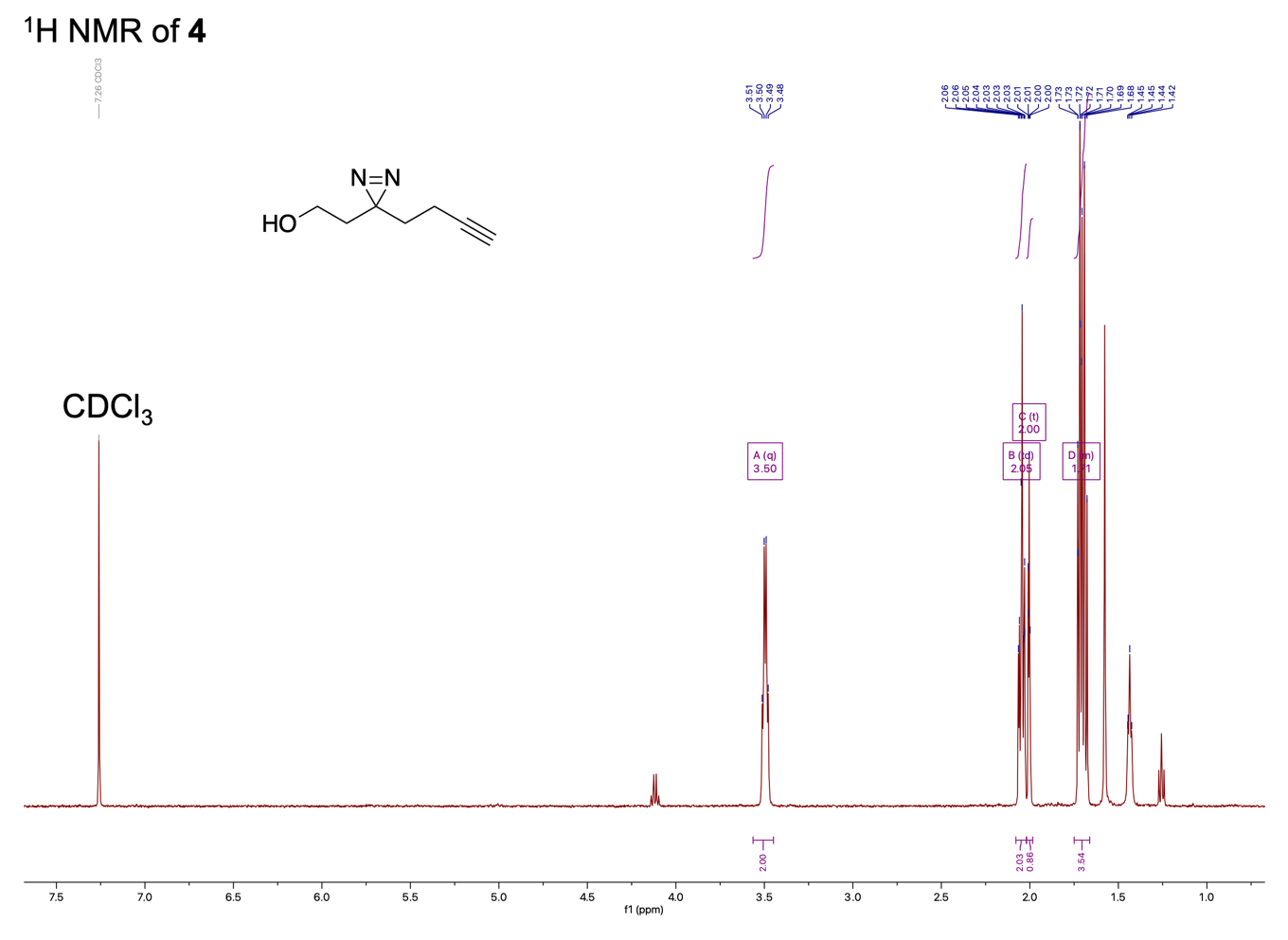
**

**
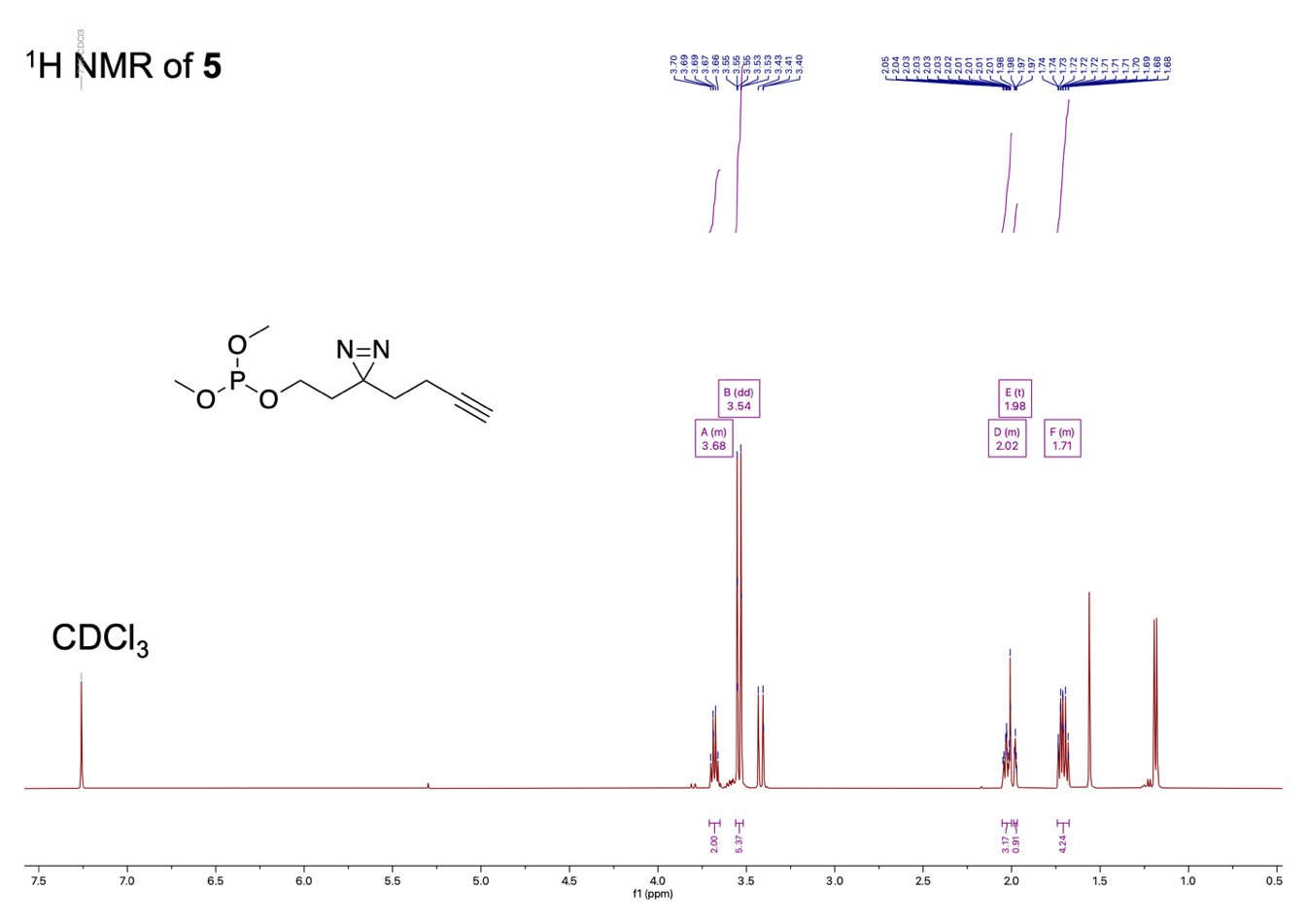
**

**
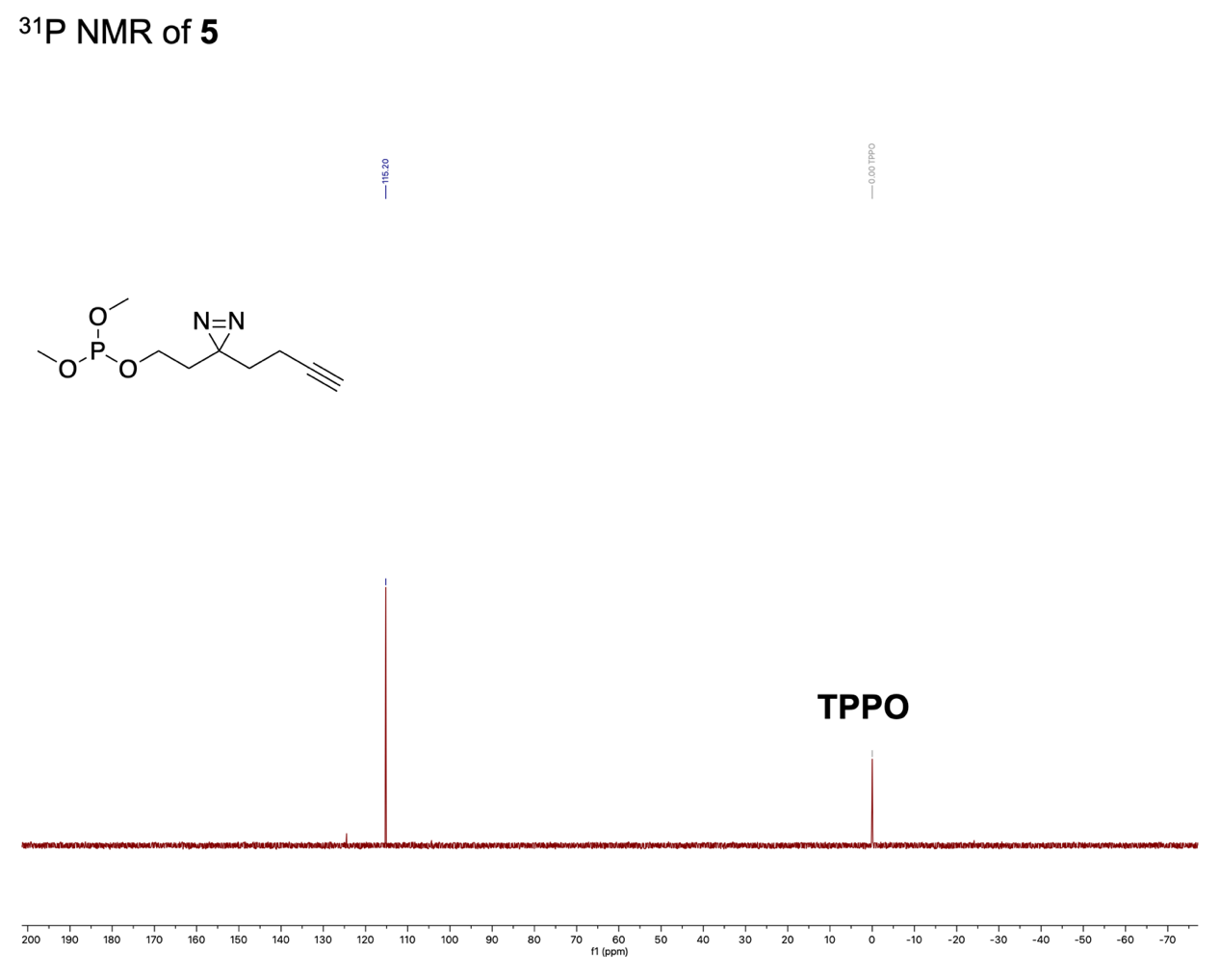
**

**3.2 High resolution mass spectrometry (HRMS) for compound characterization.
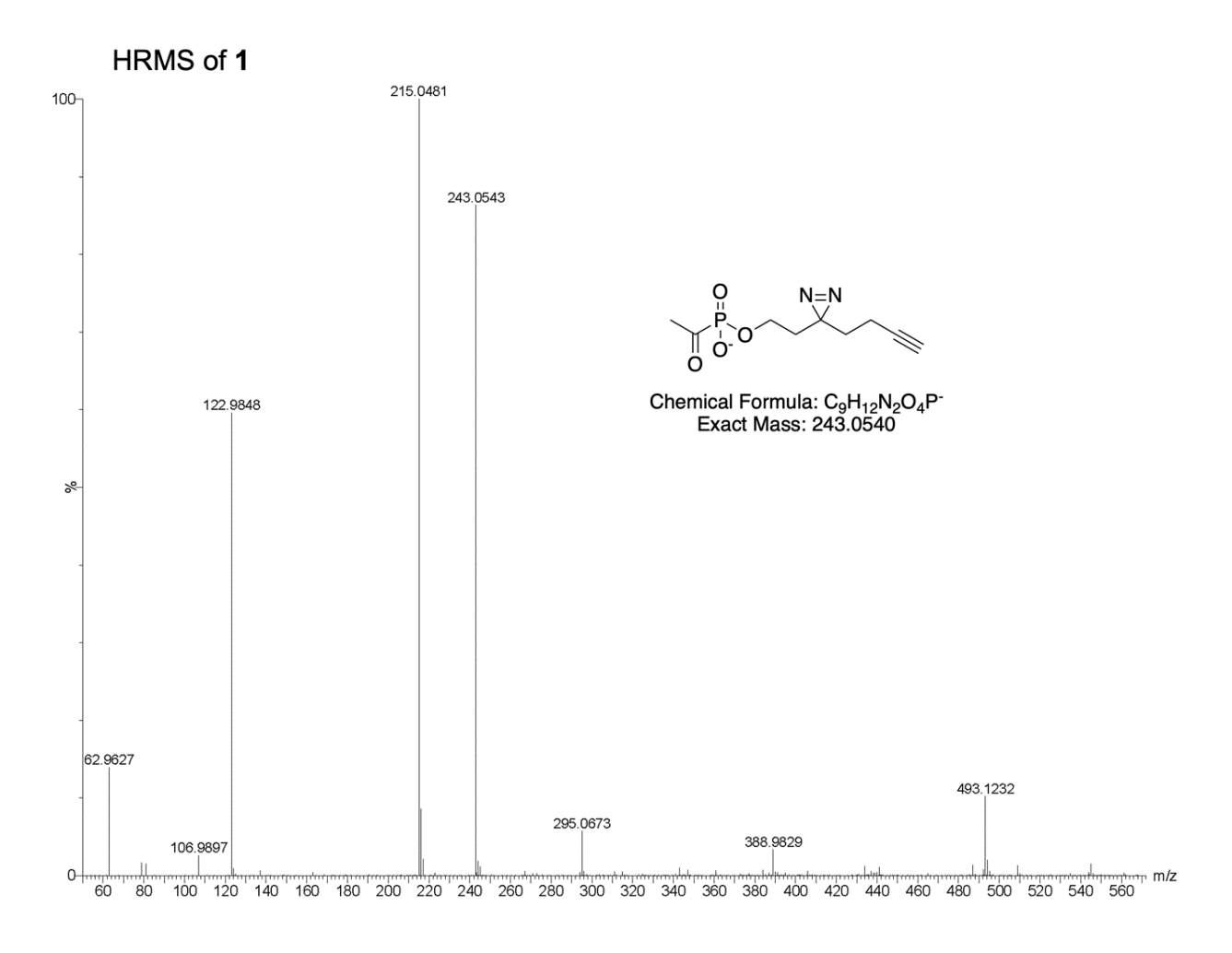
**

**3.3 HPLC spectrum for compound purity.
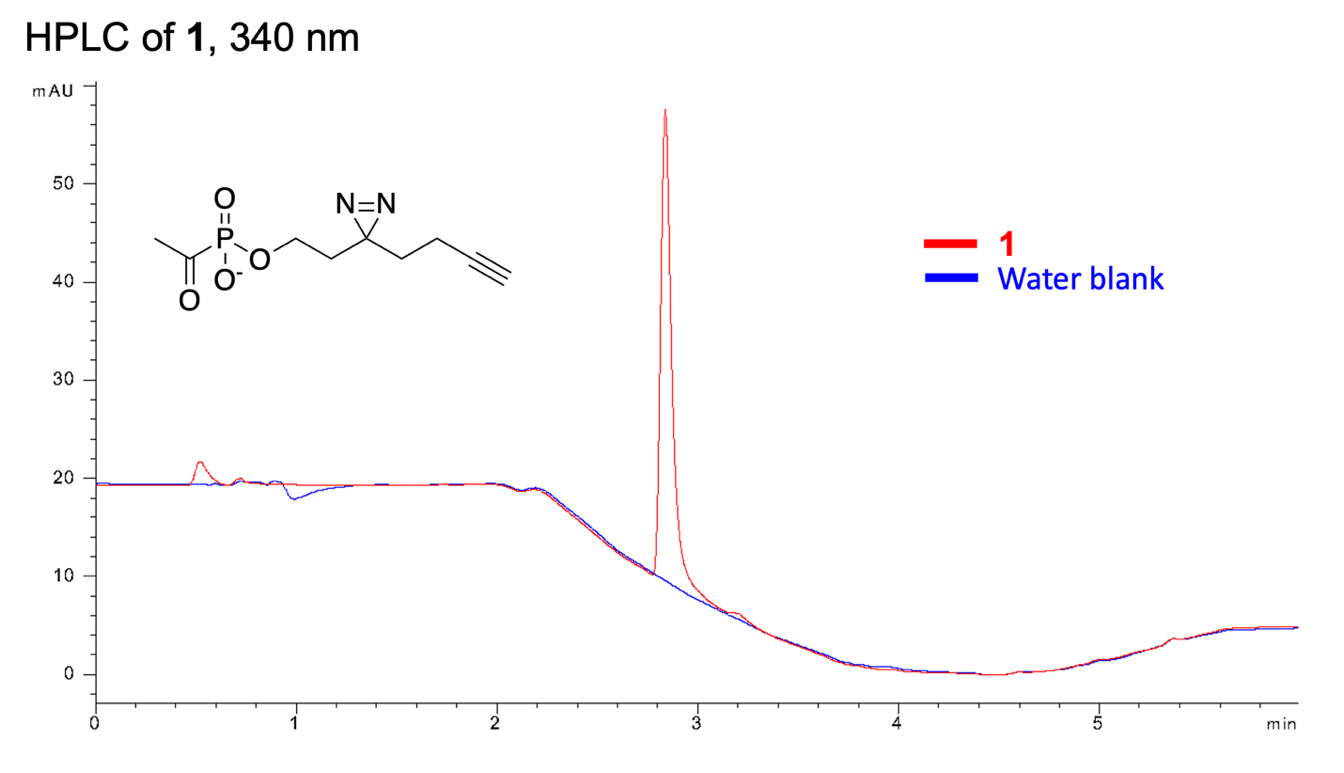
**
